# Hand Gesture Recognition on Edge Devices: Sensor Technologies, Algorithms, and Processing Hardware

**DOI:** 10.3390/s25061687

**Published:** 2025-03-08

**Authors:** Elfi Fertl, Encarnación Castillo, Georg Stettinger, Manuel P. Cuéllar, Diego P. Morales

**Affiliations:** 1 Infineon Technologies AG, 85579 Neubiberg, Germany; 2 Department of Electronics and Computer Technology, University of Granada, 18071 Granada, Spain; 3 Department of Computer Science and Artificial Intelligence, University of Granada, 18071 Granada, Spain

**Keywords:** hand gesture recognition, edge machine learning, artificial intelligence, algorithms, signal processing, radar, WiFi, 4G, 5G, LTE, ultrasound, lidar, vision, image processing, AI accelerators

## Abstract

Hand gesture recognition (HGR) is a convenient and natural form of human–computer interaction. It is suitable for various applications. Much research has already focused on wearable device-based HGR. By contrast, this paper gives an overview focused on device-free HGR. That means we evaluate HGR systems that do not require the user to wear something like a data glove or hold a device. HGR systems are explored regarding technology, hardware, and algorithms. The interconnectedness of timing and power requirements with hardware, pre-processing algorithm, classification, and technology and how they permit more or less granularity, accuracy, and number of gestures is clearly demonstrated. Sensor modalities evaluated are WIFI, vision, radar, mobile networks, and ultrasound. The pre-processing technologies stereo vision, multiple-input multiple-output (MIMO), spectrogram, phased array, range-doppler-map, range-angle-map, doppler-angle-map, and multilateration are explored. Classification approaches with and without ML are studied. Among those with ML, assessed algorithms range from simple tree structures to transformers. All applications are evaluated taking into account their level of integration. This encompasses determining whether the application presented is suitable for edge integration, their real-time capability, whether continuous learning is implemented, which robustness was achieved, whether ML is applied, and the accuracy level. Our survey aims to provide a thorough understanding of the current state of the art in device-free HGR on edge devices and in general. Finally, on the basis of present-day challenges and opportunities in this field, we outline which further research we suggest for HGR improvement. Our goal is to promote the development of efficient and accurate gesture recognition systems.

## 1. Introduction

This paper specifically deals with the recognition of hand gestures captured by a static observer. Nevertheless, it touches upon similar research areas in order to contextualize the topicamong related research topics, as well as to provide information about the origins of some of the various algorithms for successful execution of this task.

Today, an increasing number of electronic devices find their way into our daily lives. Thus, there is an increased need for novel, convenient input. The first implementations of hand gesture recognition (HGR) date back to 1993 as the paper [1] shows. Hand gestures are a common way for people to communicate. Therefore, HGR is a human–machine interface (HMI) that feels natural. There are countless application areas for touch-free interfaces. The transference of germs through vending machines or interactions with Internet of Things (IoT) devices is eliminated. A further application area is the interface with machines in sterile environments. Moreover, the advent of smart watches and virtual reality headsets demands novel input methods, as these devices have a very small display or none at all.

**Figure 1:**
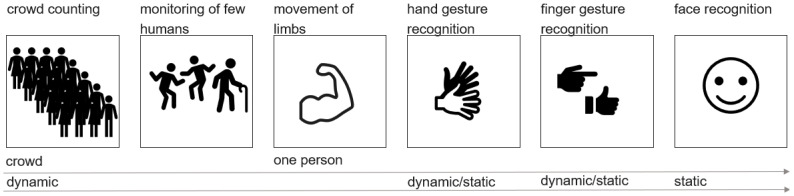
Applications of gesture recognition and similar systems.

Applications related to HGR include people counting, crowd monitoring, and surveillance of a single person. In single-person monitoring, recording very fine-grained movements is possible. This can consist of tracking movements of limbs, hand gestures, and finger gestures, or even facial recognition and vital sensing. Environment scanning, object recognition, and distance calculation are similar applictions of HGR. The goal of researching such topics can be the classification of dynamic movements or static surroundings. Many different research areas are related to hand gesture recognition. Some of them are depicted in Figure 1.

A differentiation needs to be made between hand gesture or object detection and recognition. Detection means the general existence of an object in the field of view of a sensor, whereas recognition deals with the distinction of a specific target or motion compared to others. Further, either humans or objects can be the classification target. Environment scanning encompasses both possibilities and additionally includes distance measurement and obstacle detection. Moreover, the target and/or the sensor(s) can be either moving or static, the most complicated case being when both are changing their position.

In all cases, the target has several characteristics, such as size, surface, and velocity. Additionally, the sensor itself may be moving, as in environment scanning for moving robots.

**Figure 2:**
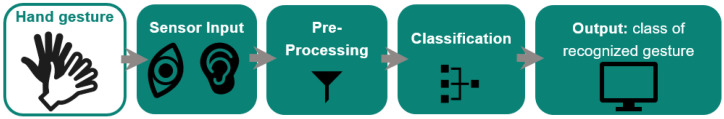
Technology-agnostic processing flow.

Upon researching the systems presented in the scope of this paper, we found that HGR systems usually consist of several common parts. The first one is a sensor type that constitutes the input method. At a subsequent stage, a pre-processing unit is used to gather sensor data and do initial preliminary processing. Pre-processing can for example comprise analog digital conversion, Fourier transform, and segmentation. In cases where classification is based on ML, data acquisition and curation are needed. Usually, pre-processing is required to prepare the data for gesture detection and classification. In a final step, the result is displayed in some form. Depending on the complexity and maturity of the technology, a single hardware can be used for only one of these steps up to all of them. More detailed comparisons of the systems are given in Figure 2, which shows the processing flow for HGR independent of the input sensor type.

Possible sensor types range from ultrasound to RGB camera and hybrid systems. A detailed description of vision, radar, WiFi, LTE, 4G, 5G, and ultrasound-based technologies, including their advantages and drawbacks, is given in Section 4.

Despite countless improvements in HGR systems to this day, many open research questions remain. Since the beginning of gesture recognition, many sensor types beyond cameras were considered. Depth measurement can be based on either camera data alone with stereo algorithm, or enhanced by an invisible infrared pattern. This is especially relevant in case of bad lighting conditions. Alternatively, depth calculation can be done with radar or ultrasound sensors and time of flight (ToF) calculation. Both technologies require significantly less power than cameras and are therefore especially interesting for the automotive or IoT sectors.

Independent of the sensor and the technology or algorithm used, some processing has to be carried out. In IoT and edge applications, processing power is scarce, as edge devices come with low processing capabilities and should use little power. Some run on battery power only. Table 1 summarizes HGR-specific challenges.

The first challenge is to accurately detect one or more hands. For a vision-based system there are tools for hand segmentation. However, vision-based systems struggle in bad lighting conditions. A similar color of a hand and the background causes further problems. Adding a depth camera can improve the accuracy of vision-based systems. In case of radar, WiFi, ultrasound, 4G, 5G, and LTE, the system relies on detecting an echo in the signal. This echo is caused by the reflection of a pulse emitted by the system from the hand. However, other objects in the field of view can also reflect the emitted pulse and produce an echo. Reflections of echoes can add further noise.

After correct detection of a hand in the FOV, the second challenge is to correctly track it. Different hand speeds lead to different gesture lengths in the time domain and different frequency shifts in the spectrogram after Fourier transformation. Normalizing and dynamic time warping are possible solutions for these differences.

The third challenge lies in gesture classification itself. The background plays a significant role in the difficulty of hand gesture classification. If it has a very similar color to the hand, classification is more challenging. The same applies for a surrounding that contains lots of noise and other obstacles that make differentiation of the hand echo from other echoes challenging. Humans have distinct ways of carrying out a specific gesture. This leads to differences in speed and location of the hand in relation to the sensor. This results in differences in gesture signals of the same gesture among individuals. Furthermore, depending on the size of somebody’s hand, the hand image or received echo signal are bigger or smaller, which causes difficulties for correct classification.

## 2. Main Contribution and Scope

**Figure 3:**
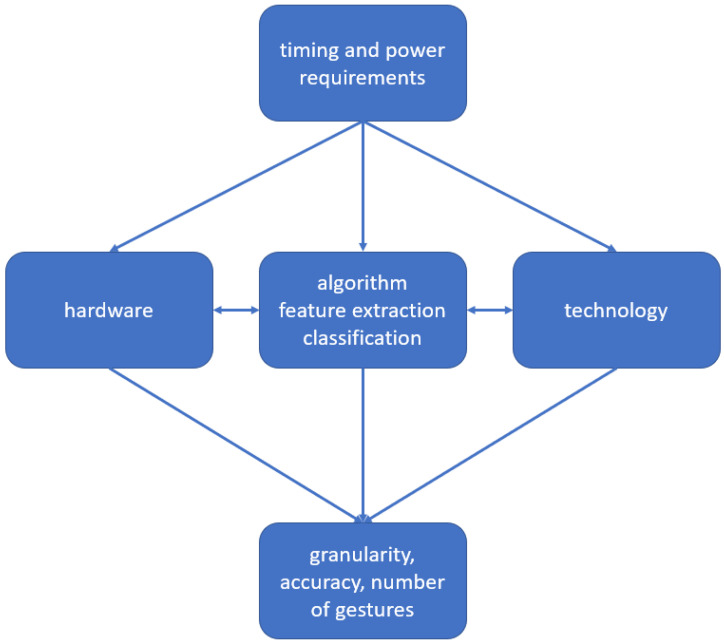
Synergy of requirements, technology, algorithms for HGR systems.

This paper explores HGR regarding technology, hardware, and algorithms. Possible application areas for HGR are wherever HMI can be found. As described in [2], this includes computer games, virtual reality, augmented reality, and health care. Choices for all three depend on the application field and beyond that are interconnected. A special focus of this paper is on which applications require which combination of technology, hardware, and algorithm, by exploring the trade-offs among them. The goal is to allow an educated choice on the best combination for a given use case. Hand gestures have different granularity and distance of the gesture from the recording device, as well as size of movement. Depending on the use case, different requirements ensue. The maximum resolution is determined by sensor technology and processing hardware. In combination with algorithms for feature extraction and classification technology, they determine which accuracy can be achieved. But technology and algorithm depend on hardware, while hardware depends on clock speed and power requirements. Figure 3 visualizes these (co-)dependencies.

Many different sensor technologies can be used for HGR, and this paper will give an overview of most of them in Section 4. Detailed descriptions of vision, radar, WiFi, LTE, 4G, 5G, and ultrasound can be found in Section 4.1, Section 4.2, Section 4.3, Section 4.4 and Section 4.5. As surveys [3,4] show, many HGR systems require a wearable sensor such as an inertial sensor or a data glove that comes with many integrated sensors. While this is an active research field, with [5] being an example of a recent paper, an assessment including those is beyond the scope of this paper. Advantages and drawbacks per hands-free sensor technology are given in the detailed description sector, and different possibilities of transmission are explored, if applicable. Section 4.6 compares HGR solutions that are based on the same type of sensor. Furthermore, information is given about which processing steps and algorithms can be applied for different sensor data types, as well as for which steps require distinct processing depending on the input type defined by the technology and pre-processing. The evaluation of the approaches described in available papers up to now shows that, in all cases, a similar general processing flow is applied, which is displayed in Figure 2. Nevertheless, the difficulty of the task depends on the used sensor type and the amount and type of gestures. Therefore, this paper uses the taxonomy given in Table 2 to categorize hand gestures.

This paper is structured as follows: The remainder of the introduction explains the focus of this survey compared to previous surveys. Then, for each technology type, an introduction of the physics and possible pre-processing algorithms is given. The paper continues with a comparison of sensor technology-dependent solutions. Subsequently, classification algorithms for HGR and the achieved accuracy are evaluated. This chapter is followed by a chapter about HGR algorithms in edge devices, which highlights work done to optimize for real-time use. The final section summarizes findings and trade-offs with the focus topics: sensor-fusion, resolution, real-time, continuous learning, robustness, no-ML, and other. Furthermore, we outline which further research is suggested for HGR improvement.

## 3. This Survey in the Context of Previous Surveys

There already exist surveys about most topics mentioned in the introduction (see Section 1), but none have specifically discussed HGR on edge devices with a focus on sensor technologies, processing hardware, and algorithms. Most previous surveys deal with vision-based systems or vision enhanced with depth information, as [6], which is an early survey about gesture recognition in vision-based systems. It already describes the general structure of gesture recognition systems which pertains until today and shows what kind of gestures were recognized before 2019, as well as which research areas gesture recognition relates to. Nevertheless, the field has evolved significantly since 2018. An even earlier publication, Ref. [7] from 2011, is about vision-based human activity recognition and describes HGR as part of such research. Another review about vision-based HGR systems is [8]. It categorizes the types of gestures into “hand and arm”, “head and face”, and “body gestures” demonstrating how diverse gesture recognition is. Survey [9] gives an overview of artificial intelligence (AI) in IoT hardware until 2020. To this end, the article evaluates edge, fog, and cloud in terms of automated perception through sensor input, reasoning, learning in a distributed network, and AI based-behavior of IoT devices in the environment.

Refs. [8,10,11] are surveys that deal with radar. Refs. [11,12,13] are about acoustic or ultrasound-based solutions. Applications enabled by WiFi are dealt with in [3]. The majority of surveys available are about vision-based systems, such as [7,14]. On the processing side, refs. [9,15,16,17] are about AI accelerators and AI in IoT. Survey [3] introduces several different sensors for HGR, but all except for the camera-based one are wearable solutions, as opposed to HGR systems considered in this paper.

## 4. Sensor Technologies

This section gives a comprehensive overview of many distinct technologies used for HGR, namely radar, ultrasound, WiFi, 5G, 4G, LTE, and optical. The different technologies are explained, elaborating on the status of current research into HGR with each of them, including an evaluation of their applicability for IoT devices. The algorithmic approach for all the technologies is outlined, with edge applications already implemented for some.

### 4.1. Vision

The beginnings of hands-free HGR are based on vision, as this survey from 2007 [14] shows. Their classification is based on a statistical model of correlation scores and dynamic time warping (DTW). DTW is an algorithm that is used up to today to account for differences in gesture speeds. Even [1] relies on a matching algorithm, which can be seen as a precursor of ML. The survey [18] shows that vision-based HGR is an active research topic up to the presnet day. The main drawback of vision-based HGR is that monocular vision does not provide depth information. Therefore, either stereo vision or ToF technology, both explained in depth in Section 5, is needed for 3D HGR. Remaining challenges for vision-based HGR are complex background, occlusion, complex hand structure, and real-time processing while preserving high accuracy. A comparison of vision, radar, WiFi, and ultrasound for HGR can be found in [19] in Table 2.

### 4.2. Radar

**Figure 4:**
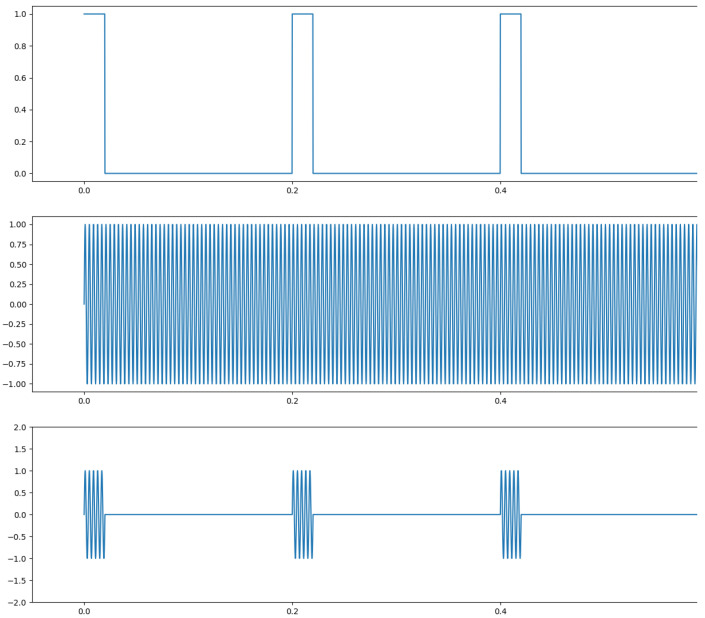
Actuation with a single frequency.

**Figure 5:**
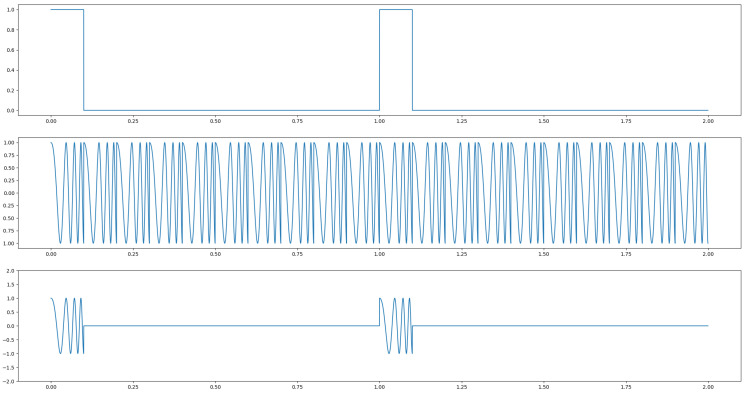
Actuation with a chirp.

**Figure 6:**
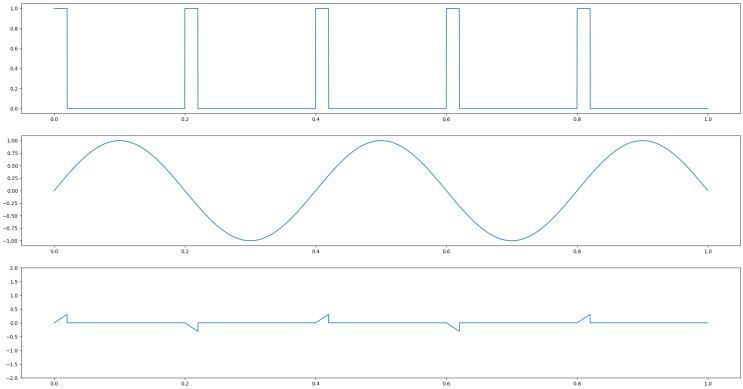
Actuation for UWB.

Extensive research has been done on radar-based hand gesture recognition. The survey [10] gives a summary of radar-based HGR systems. In radar, the send pattern can either be a single frequency or a chirp. In case of the chirp, the sent frequency is linearly increased among a given frequency band. After all frequencies have been sent, the chirp restarts with the lowest frequency. Both frequency types can either be sent continuously or as a pulse. That means the actuation of radar can either consist of a continuously sent single frequency, a pulse repeated at a given period, a pulse of a chirp, or a continuously repeated chirp for continuous wave radar. The technologies are called single-frequency continuous-wave radar (SFCW) or frequency-modulated continuous-wave radar (FMCW) for continuous actuation, and ultra-wideband radar (UWB) and Doppler-radar in case a short pulse of either one or several frequencies is sent. Figure 4 depicts an actuation with a single frequency of continuous wave, middle, or pulsed in the bottom plot. Figure 5 shows FMCW and pulsed chirp actuation. In case of UWB, a very short pulse—theoretically a dirac—is sent. A possible practical way to send such a pulse is to send a wave with a very low frequency for a very short time period. In this way, a burst consisting of many different frequencies is sent. It is depicted in Figure 6.

Radars that use frequencies in the spectrum from 30 GHz to 300 GHz are called mmWave radar, as their wavelength is between 1 and 10 mm. mmWave radar can refer to pulsed or continuous wave actuation. A frequency of 60GHz radar is commonly used for short-range applications like IoT devices and therefore HGR. The survey [10] describes FMCW, SFCW, and UWB technologies and how they are used for HGR. It gives an overview about radar-based HGR until 2021. However, especially on the algorithm side the field is rapidly developing. Radar pre-processing is traditionally based on the Fourier transform. A common way of depicting radar data is stacking actuation pulses. Applying the Fourier transform on one or both of the axes of the resulting array creates intensity maps that can be fed into ML systems for gesture classification. Possible pre-processing/map generation algorithms are explained in depth in Section 5.3 and Section 5.5. Movements in a radial direction cause changes in the frequency according to the Doppler effect, an increased frequency when moving towards the radar and a decreased frequency upon moving away from the radar. If this effect is mainly used, it is also called doppler radar. Radar is the only technology introduced here that can achieve similarly low power usage as ultrasound when the system is optimized for this goal. As it is an extensively researched technology, successful attempts with the goal of low power have already been reported, as for example in [20]. In radar HGR, oftentimes several sending and receiving antennas are used simultaneously. This technology is called multiple-input multiple-output (MIMO). A more in-detail description of this algorithm is given in Section 5.2.

The following subsections give more in-depth descriptions of common pre-processing approaches for HGR with radar, cell network, WiFi, and ultrasound waves.

#### 4.2.1. SFCW/CW Doppler-Radar

In SFCW radar, a single frequency is continuously sent and received. The received signal differs from the sent signal. All differences are caused by reflection of the sent signal at objects in the FOV. Stationary objects cause static differences between sent and received signal, while moving objects cause dynamic changes. Therefore, the received signal gives information about the FOV area of the radar. This type of actuation is simple to produce. The continuous wave creates a continuous inflow of information but requires more power than pulsed actuation. An example of an HGR based on this type of radar is [21]. It introduces a few-shot-based meta-learning system for recognition of user defined gestures based on CNN and meta-learning, feeding the algorithm with micro-Doppler images.

#### 4.2.2. FMCW Radar

This type of radar is most commonly used for HGR. The sent signal is a continuous chirp. This means that the sent signal starts at a minimum frequency and is increased up to a maximum frequency. This process is continuously repeated. The received signal is the same chirp with variations caused by the environment it is reflected off from. Those changes are what is evaluated for HGR. A range of frequencies gives more detailed information about the surrounding environment than only one frequency. So, this actuation can be leveraged for more fine-grained gesture recognition. The generation of a chirp is slightly more complex than sending only one frequency. Many researchers describe pre-processing and classification algorithms based on ML or traditional signal processing for FMCW radar-based HGR. The very recent survey paper [8] compares FMCW radar-based HGR. According to this survey, the most commonly used input for classification is Range Doppler Map (RDM), which represents speed and distance. Some papers use the angle information in addition, such as [22,23]. How those maps are computed is described in Section 5.5.

#### 4.2.3. UWB Radar

UWB radar is a type of pulsed radar where the pulse length is very small, smaller than the wavelength of the sent frequency. This creates a pulse that contains many different frequencies. A high resolution can be achieved in that way, with very low power requirements.

#### 4.2.4. SF Pulsed Radar

SF pulsed radars send a pulse of a single frequency for a time significantly longer than lambda at a given pulse repetition time (PRT). This actuation principle, like UWB radar, has low power requirements but is seldom used, as less information can be acquired from a single frequency compared to a UWB pulse and there is no continuous information inflow as in the case of continuous wave actuation.

### 4.3. 4G, 5G, LTE

HGR based on 5G, 4G, and LTE mainly uses either received signal strength indication (RSSI) or channel state information (CSI) signal. CSI contains information about rthogonal frequency division multiplexing (OFDM) for each sender–receiver pair in the network. OFDM signal can be seen as a type of frequency modulation (FM). Evaluation of this type of data, like radar and ultrasound, rely on the Doppler effect. This effect causes a frequency shift due to the radial movement of an object like a hand. As those technologies are implemented in more and more devices and their availability across the world increases, this type of HGR is a promising path. Up to now, HGR based on these communication networks has depended on the location of the sender and receiver. Their location must be known or determined before the start of gesture recognition. Mainly, gestures in radial direction to the receiver are distinguishable up to now.

### 4.4. WiFi

WiFi-based GR used RSSI to evaluate variations in signal strength in the initial stages. After a tool was developed for easy extraction of CSI, there was a transition to CSI evaluation because CSI provides significantly more information, as described in Section 4.3. Further processing is very similar to that of 4G, 5G, and LTE. Please refer to that section for a more detailed explanation of possibilities for evaluating CSI for HGR.

### 4.5. Ultrasound

In contrast to the previous sensor types, which rely on electromagnetic waves, ultrasound is a mechanical wave. Therefore, it is not influenced by electromagnetic interference. As it relies on the collision of particles, it does not work in space. In nature, animals like bats and dolphins use ultrasound for echolocation. Ultrasound-based HGR almost always works as an active sonar in air. The transmitter sends either a continuous sound wave of one frequency, or a frequency band, as in a chirp or a pulse of either one frequency or many. Humans are capable of hearing frequencies between 20 Hz and 20 kHz. Therefore, the frequencies used for HGR are usually above 20 kHz. Sometimes, frequencies between 18 and 20 kHz are used, as most humans do not hear in this range and the attenuation of ultrasound in air increases the higher the frequency. The advantage of chirps or frequency patterns is that they can even be found in case the power of the reflected echo is lower than the noise floor. Ultrasound travels with a velocity of 343 m/s, significantly slower than radio waves. For minimal attenuation, ultrasound frequencies for HGR are usually in the lower ultrasound frequency band. In that case, the signal has a lower resolution than radio-based systems. At higher frequencies, by contrast, higher resolution can be achieved than in the case of radar. The reason is that the resolution is based on a combination of the speed of the wave and its frequency. A drawback of high-ultrasound frequencies is that they are highly attenuated in air. Depending on the strength of the ultrasound source and whether beamforming is used, the maximum reachable distance for ultrasound technology is only a few meters or less. Many applications for ultrasound sensing, including HGR, are described in [13]. The authors describe how the physical effects of airborne ultrasound, for example Doppler effect and reflection, can be leveraged for different applications. This survey aims at providing further information, including algorithms used for HGR. In contrast to all other evaluated systems, ref. [24] evaluates noise created by a hand moving on a surface instead of sending a tone and evaluating its reflection. A smartphone is used for inference and the base network is ShuffleNetV2. Pre-processing is spectrogram calculation and the amount of data is increased by means of a GAN. One of the few suggestions without ML is [25]. The authors purely rely on doppler effect and thresholds for a classification of simple hand gestures. In the Section 5.1, Section 5.3 and Section 5.6 the three pre-processing methods trilateration, spectrogram, and beamforming are explained in greater detail.

### 4.6. Sensor Technology Specific Comparison

This section provides a sensor specific comparison of solutions.

#### 4.6.1. Comparison of FMCW Radar-Based Soutions

The authors of [22,23] present solutions for dynamic gesture recognition based on meta-learning, as [21] does based on Doppler-radar signals. Both use range, Doppler, and angle information as input for their system. Ref. [23] provides an extensive analysis of which algorithms for improved generalization are effective at which stage of the processing, through testing with 1-, 3-, and 5-shot experiments. Another newly emerging field is spiking neural networks (SNN) for radar-based HGR. One such approach is given in [26]. Where the authors of [26] use SNN on raw radar amplitude data. In this way the angle information is automatically retained by contrast to spectrogram based solutions like range and Doppler maps, where the imaginary part of the Fourier transform is lost. The goal of SNNs is to save computation resources. Which in combination with a high frequency of classification allows for fast and lean computation, which is crucial for real-time systems. Fine grained gestures can be evaluated using the micro-doppler effect. The spectrogram, is usually extracted from a received FMCW radar signal. Papers [27,28] describe such an approach. Ref. [27] leverages orthogonal matching pusuit (OMP) algorithm for extraction of features from the micro-doppler map. Those features are used to choose one out of 4 possible fine-grained finger gestures with the modified Haudorff distance classifier. Ref. [28] uses radar data to distinguish 1 or 2 person/s walking from a wheeled vehicle at high distance, with low resolution with micro-doppler features and multi-class SVM.

#### 4.6.2. Comparison of UWB Radar-Based Solutions

The authors of [29] introduce a system using one or two UWB radar/s in combination with GoogLeNet classification algorithm for feature learning and classification of eight hand gestures. The used radar is Xethru X4. The center frequency is 8.748 GHz, with a bandwidth of 2 GHz. The duration of each gesture is 5.5 s. The accuracy achieved was 91.25% with one and 93.75% with two radars. Another UWB radar-based approach, presented in [30], compares the classification accuracy of four different algorithms for classification of 14 gestures. The features fed to the algorithm are in this case range-Doppler frames. The radar used is XeThru X4 Radar, as in the previous paper, with center frequency 7.29 GHz and bandwidth 1.5 GHz.

#### 4.6.3. Comparison of 4G, 5G, LTE Based Solutions

The authors of [31,32] describe two approaches for 5G-NR (New Radio) and G4 based HGR. In [31] a system is introduced that uses the CSI (channel state image) and the Doppler effect to recognize gestures. The authors of [32] describe a LTE based systems. They describe their findings about the dependence on the direction which corresponds to the location of the hand gesture in relation to sender and receiver. As HGR highly depends on that position they found a solution as to how to discern the location of the hand as a first step such that the movement can be done in the right location. In article [31] the HGR system is based on 5G-NR (New Radio) waves. As 5G coverage is expanding, HGR on this type of network will likely become increasingly interesting in the future. Even in this pioneer paper a high gesture accuracy of more than 90% could already be achieved. It additionally shows that a higher bandwidth allows for higher accuracy values. Paper [33] shows a real-time approach for HGR with CSI of LTE signals without ML-based on DTW with similarity measure.

#### 4.6.4. Comparison of Wifi-Based Solutions

One of the latest approaches [34], suggests to use similarity decay between CSIs with distance. Two setups are evaluated, an office and a home environment. For the first, a setup of three, for the second, a setup of two transducers is placed in an apartment. The gestures are detected even through walls. The classification consists of the detection of the beginning of a gesture and continues with an extraction of primitives consisting of rising and falling edges in the RSSI signal. A sequence of primitives makes up a gesture. In [35] several uncorrelated WiFi senders are used as signal source. A sparse array works as receiver. It has to be mentioned that in both cases the setup and the signal transmission is not as in usual WiFi signal communication. The survey paper [3], is about usages of WiFi in general, among them HGR. This task is carried out based on RSSI for coarse grained gestures and CSI for more fine-grained gestures, including finger gestures, and keystroke recognition. Furthermore, Doppler shift is presented as a possible base for classification of 9 arm or leg gestures.

#### 4.6.5. Comparison of Ultrasound Beamforming Solutions

The authors of paper [25] positioned an array of eight transducers in the upper left corner of the phone. Combined with the phone’s built-in loudspeaker, these transducers serve as the system’s hardware. They chose this placement to minimize user disturbance. In both directions Delay-and-Sum beamforming is used to evaluate the data before it is pre-processed into a spectrogram that is evaluated by a TCN. Paper [36] is a HGR system based on distance calculation on an dedicated hardware, with extremely small power requirements. At a FoV of ± 45° and at a distance of between 4.5 cm to 1 m. The output of the system is an indication of size, distance and location of objects in the FoV. Ref. [37] uses one loudspeaker and eight microphones that surround the loudspeaker. The evaluated features in this case are distance, derived from the peak of the data in time and the intensity of the signal in the surrounding of the peak. Evaluation is done by CNN with LSTM for two to five gestures. This paper is one of the few that compares their system’s performance for different numbers of gestures. Ref. [38] uses custom hardware of seven receivers and one transmitter for object localization based on beamforming in a FoV of 90° within a distance of 1 m. A finite state machine can be used to do gesture recognition as the location of an object like a hand directly allows the evaluation of changes of the position and thereby a gesture. Gesture detection is done by evaluating if an object is being found in a specified start area in the FoV. Swipe gestures can successfully be recognized in this way. A hexagonal array of six transducers, with one sender in the middle is used in [38], to do Doppler-shift evaluation at different angles for dynamic gesture recognition. The classification is carried out in a very processing power efficient way with a TCN. Ref. [39] combines a big piezoelectric transmitter with a circle of eight MEMS receivers. Using a processing combination of DOA estimation with a search algorithm and delay and sum beamformer. Only locations where both algorithms return high values are kept. In this way several static objects can be detected. Ref. [40] relies on an array of 64 transducers in a square arrangement for the creation of an image of the surrounding. As the transducers used are rather large, a waveguide is required to achieve the benificial $\frac{\lambda }{2}$ distance. Sender side beamforming as well as beamforming only on the receiver side are evaluated. The system works at 40 kHz. Ref. [41], like [39], it utilizes a small amount of transducers. Different arrangements were theoretically considered. The outcome was that a square arrangement is most promising. The software approach consists of a mix of sound source localization with MUSIC algorithm and minimum-variance-distortionless-response (MVDR) beamforming. By contrast to [39] an array is also used on the sender side, not only on the receiver side. The authors of [40] also published about a fermat spiral based arrangement of their 64 transducers and developed a sophisticated sender and receiver side approach. All four, Refs. [39,40,41,42], focus on scanning the surrounding. This task is different from dynamic hand gesture recognition, in that it scans static objects rather than moving objects. The approach presented in [43] uses receiver side delay-and-sum beamforming in both direction along the l-shaped array. The transmit pulse is sent from a different loudspeaker at a distance of several centimeters. The classification is done with a TCN on the 1D time domain data. Ref. [44] evaluates the three different beamforming methods MVDR, Delay and Sum, and Filter and Sum, for receiver side beamforming in combination with an omnidirectional sender. A TCN is used for classification. This approach is similar to DoA estimation and evaluation of frequency shift, which allows for classification of tap gestures from different angles. Source [45] compares several fermat spiral arrangements of ultrasonic transducers to verify which one has the best beamforming characteristics. Tese are good side lobe suppression and a precise main beam. A comparison of spiral and square arrays is done in [36]. In [46] a linear array of $\frac{\lambda }{2}$ is used for distance estimation of several static targets at different distances and directions, including angle estimation. The same authors also successfully investigate an 217 kHz array for the same purpose, as in [36]. The choice of frequency is a trade off between angular resolution and distance. A higher frequency causes increased attenuation but at the same time better angular resolution. As at some point $\frac{\lambda }{2}$ is too small for the diameter of the transducer, in the second case the receivers are arranged in zig zag arrangement.

## 5. Pre-Processing/Signal Processing

This section introduces pre-processing methods used for HGR. Multilateration is used for distance calculation, beamforming to enhance the SNR level, and Fourier transform to evaluate the signal in the frequency domain. For ultrasound data processing and evaluation of fine-grained radar data the spectrogram is usually calculated. In the radar domain it is often called micro-doppler. Especially in the radar domain Fourier transform is used to generate data representations as RDM, ADM, and RAM.

### 5.1. Stereo Vision

Stereo vision is the calculation of a depth image based on two images of the same scene from different angles. It works like human vision. Objects in one image are at a different location in the second image. This difference applies to all pixels of the image and is called disparity. On the basis of this disparity a 3D point cloud can be constructed. Stereo vision is often used in combination with object detection algorithms to enable object tracking.

### 5.2. Multiple-Input Multiple-Output

Multiple-input multiple-output (MIMO) is based on the fact that a signal sent from different locations follows a different path even though the same receivers are used. Two senders and two receivers therefore produce four distinct signals that each contain different information and is a virtual array of four. When using one sender and three receivers we would only get the information from three receivers. Each transmitter doubles the output of the receivers. This technology is mainly used for improved angle resolution, calculated using the phase change between the signal coming from the senders.

### 5.3. Spectrogram and Micro Doppler Map

**Figure 7:**
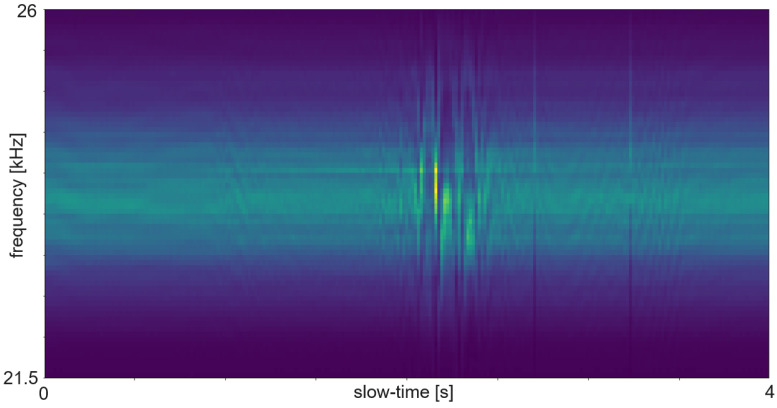
Spectrogram (in radar often called micro-doppler), calculated by 1D Fourier transform in fast-time direction, over the entire sample in a frequency band around the sent single frequency.

The term Micro Doppler Map is often used instead of spectrogram in radar processing. In most cases the spectrogram is calculated such that the time bins are as long as the pulse repetition time. Sometimes the time bins are a bit longer. The additional time causes an overlap between the bins and consists of more actual data or added padding. Figure 7 shows an example of an ultrasound spectrogram. The unique doppler shift of a moving hand in the FoV of the ultrasonic transducers can be used for gesture classification. An exemplary use case of micro-doppler on radar data are fine finger gestures as described in [27]. Another application is differentiating distinct types of moving targets at high distance with low resolution as in [28]. Spectrogram evaluation is used for fine-grained gestures in radar or for the evaluation of ultrasound data.

### 5.4. Phased Array/Beamforming

**Figure 8:**
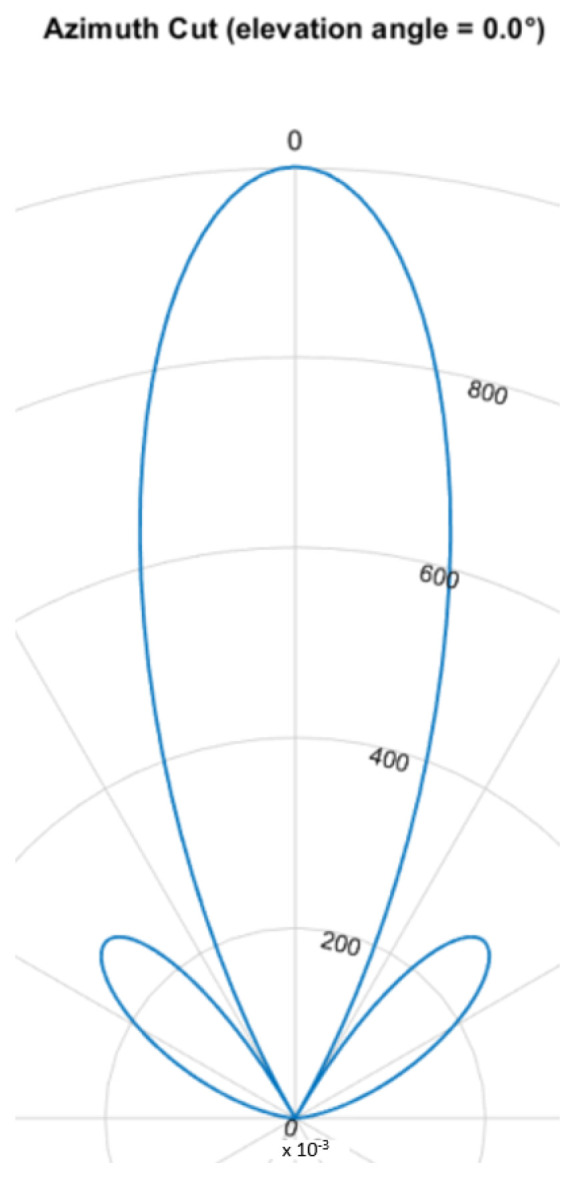
4 ultrasound sender at $\frac{\lambda }{2}$ spacing, 24.5 kHz frequency, 0° steering.

**Figure 9:**
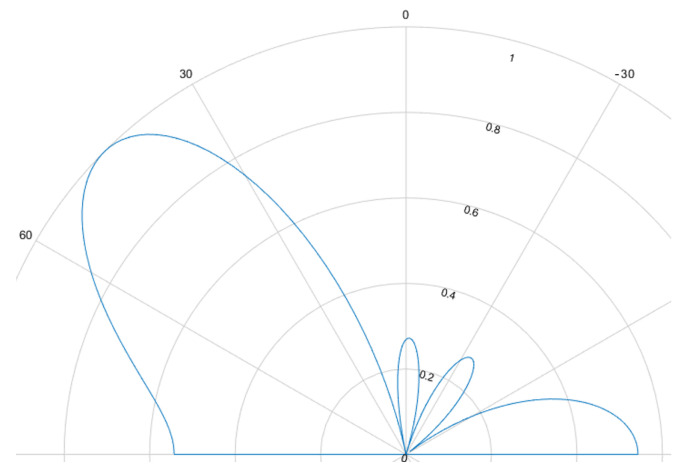
4 ultrasound sender at $\frac{\lambda }{2}$ spacing, 24.5 kHz frequency, 45° steering.

**Figure 10:**
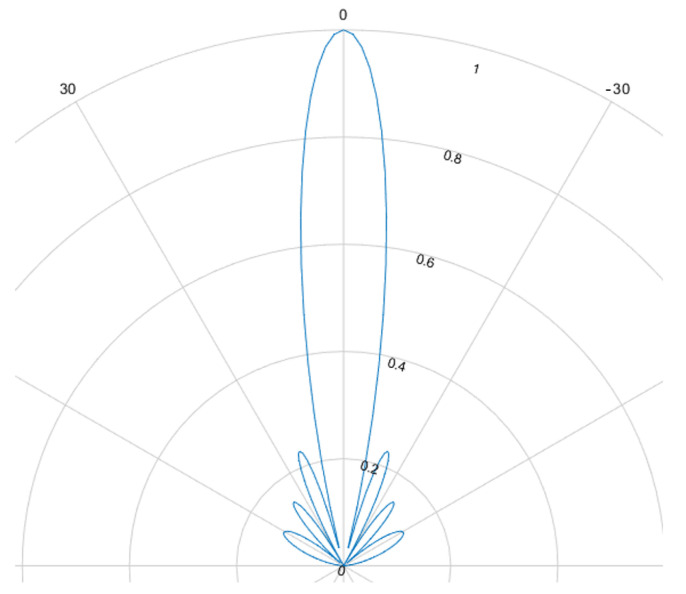
8 ultrasound sender at $\frac{\lambda }{2}$ spacing, 24.5 kHz frequency, 0° steering.

**Figure 11:**
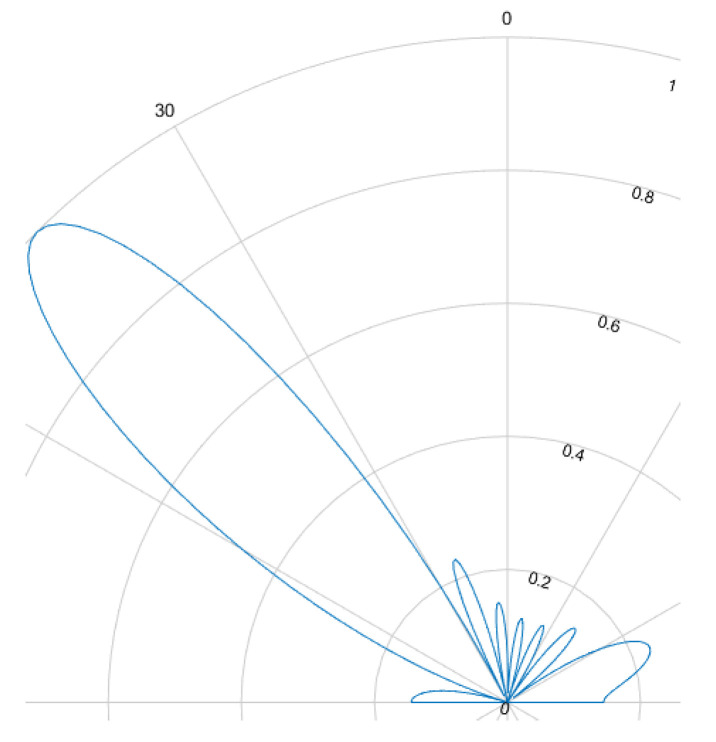
8 ultrasound sender at $\frac{\lambda }{2}$ spacing, 24.5 kHz frequency, 45° steering.

The beamforming effect is caused by overlapping signals interfering constructively or destructively. This interference is either used to focus and steer an emitted beam or to determine the direction of arrival. For ultrasound HGR the far field assumption applies. Most cases rely on delay and sum (DaS) beamforming but MVDR is also possible. Figure 8, Figure 9, Figure 10 and Figure 11 show the simulated steering capability of a linear array of four and eight ultrasound transducers at 0° and 45° with DaS beamforming. The steering capability is dependent on the number of transducers in the array, as Figure 8 and Figure 10 show. The fewer tranducers are used the wider the main beam.

For DaS beamsteering, the direction of a beam is determined by delaying the signal of the senders such that the signal positively interferes in the desired direction while other directions, especially surrounding the desired direction, are locations of destructive interference such that the actual signal is only sent in the desired direction. Through constructive interference the signal is multiplied which leads to higher possible distances and stronger received echoes. A drawback of this method is, that a smaller area can be evaluated one at a time. If beamforming is applied on the receiver side, the signals of several receivers are shifted in time and summed to cause the same effect. Thanks to parallel and multi core processing many directions can be evaluated at the same time. DaS beamforming is a form of fixed beamforming. It has no feedback loop to take the current environment into account. MVDR beamforming by contrast is an adaptive beamforming method. That means it depends on weights that are adjusted depending on the noise environment. If unwanted noise is detected weights that correspond to that directions are lowered to disregard the information from that direction in further processing of the signal.

### 5.5. Range-Doppler Map, Range-Angle Map, Doppler-Angle Map

**Figure 12:**
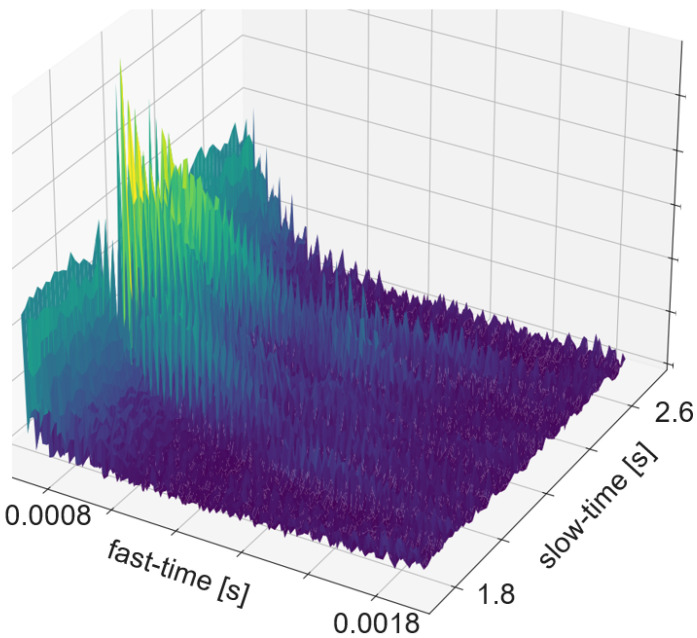
Raw ultrasound data sample in 3D.

**Figure 13:**
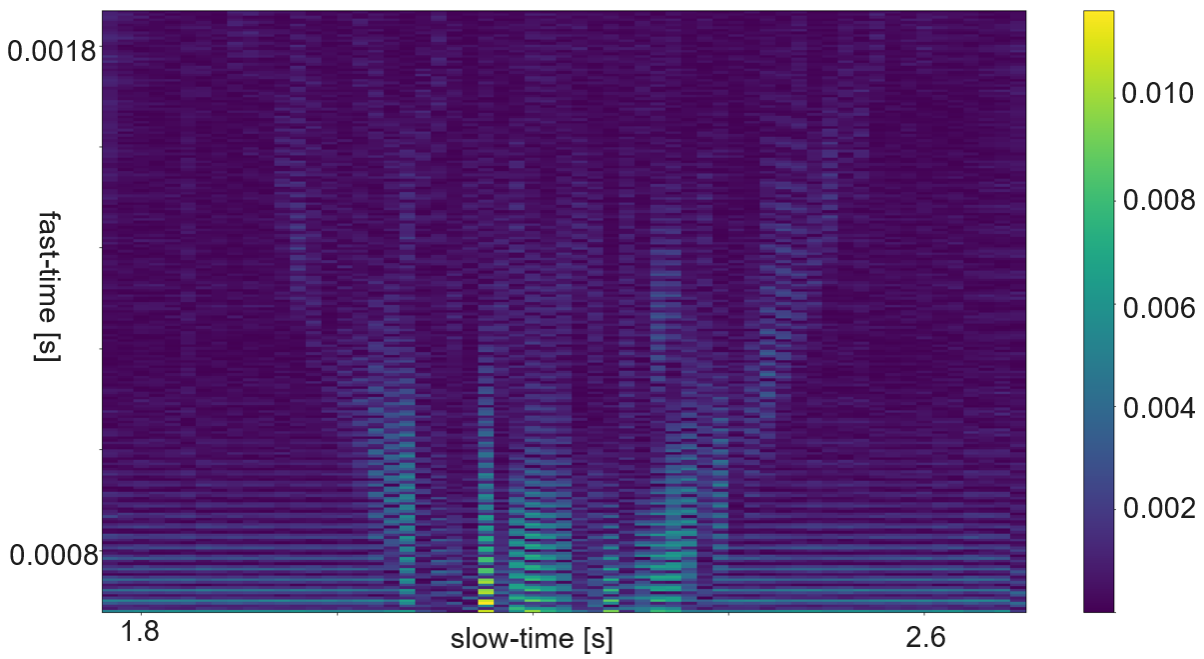
Raw ultrasound data sample in 2D only segmented part plotted.

**Figure 14:**
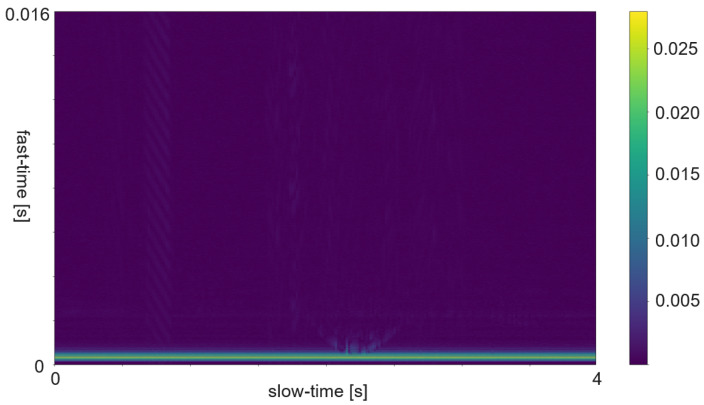
Raw ultrasound data sample in 2D.

**Figure 15:**
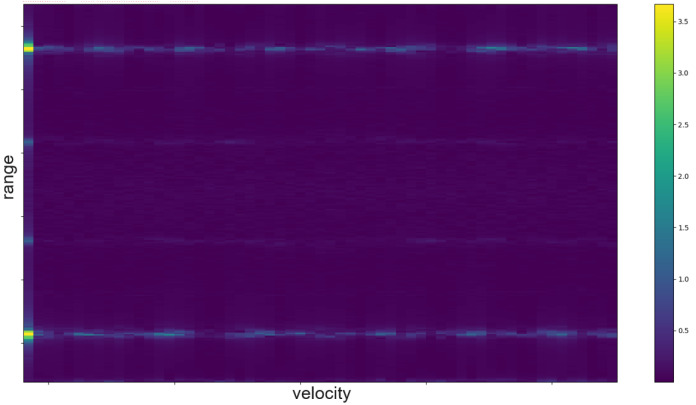
Range-doppler/velocity map, calculated by 2D Fourier transform (in fast - first and then in slow-time direction), of the segmented gesture.

The RDM is a matrix or an image created by applying the 2D Fourier transform to the received data. In a first step, the raw data is depicted as an array. That creates a matrix of amplitude values. On the x-axis each step corresponds to one data frame. Each frame corresponds to one pulse or chirp. The data along the y-axis represents the values gathered within one frame. Thus, each step on the y-axis is a time step of the sample frequency. The Fourier transforms for range are in the direction of slow-time and for Doppler in the fast-time direction. Doppler means the frequency shift through Doppler effect caused by an object that moves towards or away from a frequency source. Angle is estimated by evaluating the phase shifts between several receiving antennas. As the distance among them and the distance between receivers and sender are known this allows to calculate the direction of an object. A segmented sample of an ultrasonic push-pull gesture is depicted in Figure 12, where the z-axis corresponds to the amplitude values. The same data is plotted in 2D in Figure 13. Figure 14 shows the full sample. We can see that the segment zooms into a part in the middle, bottom of it. Figure 7 is derived through application of one Fourier transform in the fast time direction. That means along each pulse. Finally, Figure 15 shows the actual RDM. As the input to the Fourier transform is real data, the image is symmetrical. Micro-Doppler maps are spectrograms. Both represent frequency components of a signal over time. This type of pre-processing is used to evaluate smaller displacements sometimes on top of a large movement with higher granularity.

How closely related the topics of radar and ultrasound-based HGR are shows in the interrelation of papers [8,47,48,49]. Ref. [47] is an extension of [48], which is based on [49]. And [49] uses the same features as the authors in this FMCW radar application [8]. The features were selected according to a feature selection algorithm described in [8]. All systems are based on pulsed actuation. In case of radar the pulse consists of a sweep, for ultrasound one constant frequency of 40 kHz is being sent. In all cases the first step is to calculate the RDM. This pre-processing consists of several steps. First, the amplitude values are divided in the pulse trains and stacked such that the pulse trains are represented in x-direction and the y-direction ticks are steps from one pulse train to the next. Therefore, the x-direction is called fast time and the y-direction slow time. The following steps are an FFt in x- and in y-direction. The change in frequency along fast-time represent velocity of an object perpendicular to the sender, while changes in frequency among consecutive pulses represent distance changes. The creation of a RDM is explained in more detail in Section 5.5. A range-doppler image of ultrasound sensor data is depicted in Figure 15. As one of these maps can be created per sensor, stacking temporarily connected maps creates a data cube. Differences between the maps of several sensors add information about the location and direction of travel of objects in the field of view.

### 5.6. Distance Calculation and Multilateration

Distance calculation uses the knowledge of the speed of propagation of either sound at 343 m/s or electromagnetic waves such as radar, WiFi, and light of 299,792,458 m/s.

High pass filter, cross-correlation, time gain compensation, and summation of several pulses are common algorithms to find the echo in the received signal. An especially positive effect of cross-correlation is, that it lowers the length of the echo while the center points of the echo are elevated. In radar processing these steps are usually carried out inside the radar unit. For ultrasound the sensor hardware usually outputs the amplitude of the sound pressure level directly. Due to the lower frequency of ultrasound the distance calculation steps require less processing power. Beamforming in combination with distance measurement results in knowledge about the angle of arrival (AoA) and distance.

While one sensor suffices for distance calculation, at least two are required for multilateration. The receivers should be placed at a distance significantly higher than $\frac{\lambda }{2}$. For omnidirectional sensors each distance measurement of a sensor results in a sphere of that radius of possible locations for the detected object. The object of interest must be located, where the spheres of possible distances measured by several sensors intersect. In case of several intersections usually some can be excluded due to the FoV of the sensor or other given restrictions, like for example the movement range of a hand. Theoretically distance measurements to three sensors allows the exact localization of a point object. But in reality distance measurement is not perfect and a hand is not a perfect point object. Additional measurements can compensate for noise in combination with statistical methods like maximum likelihood estimation.

## 6. History and Categorization of Existing Approaches

**Figure 16:**
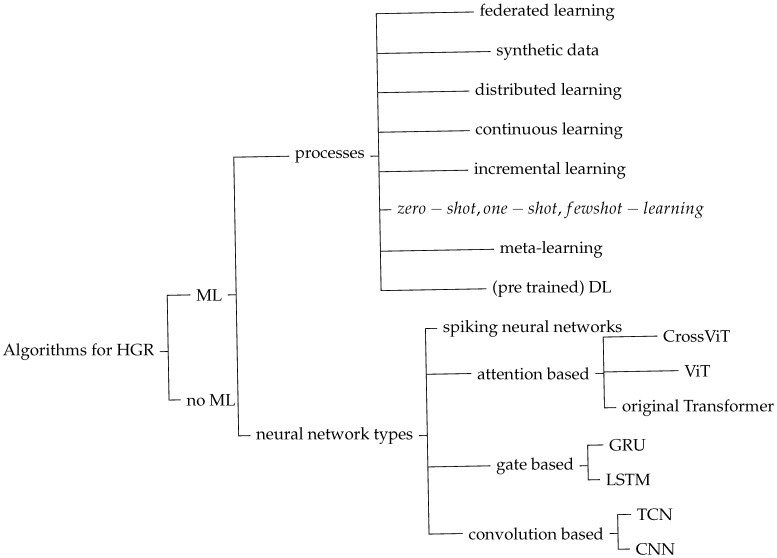
HGR classification algorithms and processes.

This section introduces diverse processes and algorithmic approaches for HGR. Figure 16 depicts an overview of these processes and algorithms. First of all, certain HGR algorithms are based on ML, while others are not. Depending on whether machine learning is applied, the entire setup, development, and assessment process is different. ML-based algorithms can use different processes and implement distinct neural network types. The processes are listed as child nodes to the right of processes. Available neural network types used for HGR are based on specific principles: spiking, attention, gate, and convolution. Child nodes to those network principles show variances of each of them. Each variance serves a specific purpose. More detailed information about the neural network types and their variances can be found in Table 3. A summary of advantages and drawbacks of different sensor types is given in Table 4.

HGR began with vision-based systems such as [50]. This system uses vision-based depth calculation and feature extraction without ML. It was already published in 1998. Ref. [51] is also one of the earliest works about HGR, being published in 2005. This vision-based system still comes with many constraints. The system presented in [51] requires the user of the system to wear a glove and to keep their hand still at the beginning of the gesture. In 2015, Ref. [52] presented one of the earliest hand gesture recognition systems in radar. Ten hand motions and a thumb motion are recognized using radar data in the system that is being displayed. To evaluate the system the detected coordinate was compared to one measured by a depth sensor. At about the same time the first ultrasound-based systems like [53,54] emerged, which classify several hand gestures using the audio system of commodity devices like a laptop or a smartphone respectively. First attempts to use WiFi for HGR have also been made, as shown in [34]. Despite of reseachers’ efforts since the 2000s, even today HGR systems still require improvements in terms of robustness to interference, environmental changes, different users and similar.

The latest systems rely on combinations of classification algorithms. LSTM and GRU structures are well suited for time series data as hand gestures. Transformer is a structure that allows for time-sensitive processing through positional encoding. Ref. [55] uses a combination of CNN and Transformer to create robust classification in the face of interference from for example an object being thrown in close vicinity. Ref. [20] presents a system that is specifically developed to fit on an Arm Cortex-M4 microcontroller. Five features, magnitude, horizontal and vertical angle, velocity, and distance, are used in this case to classify using an RNN structure.

## 7. Comparison of Algorithms for HGR

A wide variety of algorithms are useful for HGR. Convolutions, gate mechanisms, tree structures, or transformer structures are the foundations of the algorithms. Some use pre-trained deep networks that are accessible online. Implemented machine learning techniques include meta-, few-shot-, ensemble-, and semi-supervised learning. Supervised learning was used in the majority of cases. Nevertheless, Refs. [39,54] demonstrate, that signal processing without ML and unsupervised algorithms can be successful. The presented solutions have different focus. Solutions with a completely new approach usually show a prove of concept that HGR is generally possible with their novel setup. The approach’s advancement can either be on the algorithm side, on the hardware side, or a combination of both. The improvement is either focused on reducing required resources like power, processing steps, and time as in [20] or on increasing accuracy or robustness of the system, as in [56]. The drawbacks of ML algorithms are requirement of a huge, versatile data set for training and being dependent on the setup of the system. Sometimes even on the specific sensor hardware and environment used. Un-supervised training with new data collected while the system is already in use can be a solution to this problem. If this is done automatically it is called continuous learning. Furthermore, this allows to adapt the system to the current user, setup, and surrounding, improving robustness and accuracy. Zero-, one-, and few-shot learning allows users to add new gestures on the go. Since the early days of convolution based neural networks in the 90s, starting with the introduction of LeNet in 1998 for digit recognition in images, this network type kept evolving and is nowadays used for many tasks including HGR. It soon became obvious that a trade off between network depth and efficient processing must be found as [57,58] show.

The algorithm presented in [37] relies on a circular arrangement of one ultrasonic sender and eight receivers whose data is being evaluated with a combination of CNN and LSTM. Input features are either depth or intensity, or a combination of both. CNN accounts for space and LSTM for time evolution of the gestures. In [59] a ten layer CNN is implemented for HGR with radar micro-doppler maps as input. The authors of [60] extract changes in ultrasound CSI and create a tensor containing space, time, and number of receiver microphone to use it as network input for a CNN model. Ref. [22] introduces the use of meta-learning for HGR. For meta-learning two models are trained. One is designed to help in the learning process of the other. The first one is trained to discover for example the best initialization values for the hyper-parameters of the second one. Utilizing the output of the first one, the second one, which is designed to solve a specific task, requires significantly less training data and time. In the case of [22], the task is HGR. The data is given in three different styles: range-frame-map, doppler-frame-map, and angle-frame-map. Parallel processing of the three input modalities and merging the result gives the highest overall accuracy. Ref. [44] uses a hexagonal arrangement of seven ultrasound transducers. For the processing, beamforming, feature extraction, and a temporal convolutional network are applied to ultrasound spectrograms. The authors of [25] base their predictions on ultrasound data collected with a Samsung Galaxy S10e phone’s top and bottom speaker and evaluate hand gestures with Google’s Xception model, compare it to a six layered CNN and evaluate late and early fusion. The authors of this research have published their data set. Ref. [21] introduces the use of few-shot learning with one or three support samples for convolution based HGR with micro-doppler radar data. That means, a new, unknown gesture can be performed by the user either one or three times, to make that gesture known to the system which is then able to recognize it. The algorithm consists of an encoder module for automated feature extraction, a comparison model to assess the similarity of the extracted features, and a weighting module that evaluates the similarity measure. The embedding—and the comparison module carry convolutional layers. In [61] all received inputs are initially processed separately with 1D convolutional layers and inception layers. The actual classification uses an LSTM layer. The authors of [62] leverage a combination of deep CNN with the encoder part of transformers and adaboost for ensenble learning on WiFi data. Their main goal is to improve cross domain reliability of the system. The focus of [63] is on edge implementation. Their CNN therefore only consists of feature extraction on each channel, linear transformation, and point convolution, while the main part of the system is used for feature extraction from WiFi data. Ref. [64] found that CNN yielded best results compared with LSTM and decision tree classifiers for gesture recognition on WiFi data.

Gestures recognized with WiFi are more coarse than those recognizable with vision, radar, or ultrasound technology and rely on CSI.

The following papers deal with convolution based HGR since 2023. Ref. [56] implements a deep CNN on the complex data generated using FFT on MIMO (Multiple-input Multiple-output) radar data. By contrast to the previous approaches the imaginary part of the data is used to increase accuracy. Ref. [65] Generates huge amounts of artificial data using GAN for improved training of a CNN on ultrasound data. Applying a mix of supervised and unsupervised learning, Ref. [66] enhances HGR’s cross-user and cross-environment classification outcomes on radar data. The convolution-based model is trained with labeled and unlabeled data with labels generated by a guessing algorithm. Additionally, it compares pre-processed data from Range-Doppler-, Range-Angle-, Doppler-Angle-Image, and Micro-Doppler Spectrograms, showing that RAI performs best in these comparisons. Ref. [67] employs a combination of convolution and gate based (LSTM) model for HGR of two persons simultaneously with one radar. CNN and TCN are combined in [68] for radar data-based HGR. Ref. [55] is also based on radar data and uses a combination of CNN and transformer for classification of hand gestures. The classification solution presented in [69] is purely gate based, implementing two LSTM layers for the HGR task on radar data. The setup presented in [70] is adjusted for being as power efficient as possible. Therefore, it bases its classification on a spiking beamforming algorithm for AOA estimation in a first step and a lean spike-based RNN for classification. Ref. [71] actuates a big membrane to create a huge spectrum of ultrasound frequencies, which are evaluated for HGR based on two GRU layers. Ref. [20] targets to implement a real-time system for HGR based on radar data on an ARM Cortex-M4 device using a small RNN for processing.

## 8. Efforts to Bring HGR to the Edge

The survey [72] is the first to attempt a standardization of the definition of edge AI. To this end, it distributes the aspects of edge AI into four main sectors. The first one is fog computing (FC), mobile edge computing (MEC), micro data center (mDC), and cloudlets. FC describes the insertion of a field area network between sensors and the cloud. The field area network allows interconnection and “intelligence” close to the sensors and controls the communication with the cloud, while the sensors are connected to the field area network. The idea behind the MEC paradigm is to leverage the radio access network as a network to connect sensors close to the edge. mDC focuses on applications in the context of Industrial Internet of Things (IIoT). In case of cloudlets, several mobile devices would connect to create a network close to the device itself with increased computing power, only connecting to the cloud when required.

Making an algorithm edge applicable always goes along with decreasing the memory footprint, lowering processing latency, and power consumption. In most cases, only inference is carried out on the network edge. That means the previously trained model is deployed on the edge to carry out predictions but no actual training takes place in the edge node. A possible way to leverage edge nodes for training is ensemble learning, where the training either takes place in sequential steps, each improving the previous model, or in parallel on different data sets, only combining model parameters to a final shared model with improved accuracy and robustness. But no algorithm based on ensemble learning was used by the considered sources for HGR.

Table 5 and Table 6 give a detailed comparison of all real-time systems discussed in this paper. All edge implementations are also real-time systems. The column “Effort for edge applicability” states which steps were taken to speed up the system and lower resource requirements. Not all of these efforts lead to edge applicability. The approach of [40] still requires a GPU. But the processing requirements highly depend on the type of sensor used and the goal of the paper. While some see edge applicability as their goal, others focus on robustness. Furthermore, hand tracking is significantly more processing intense than classification.

Specific optimized implementations are presented in [38,43,63,73]. Some of these even run on FPGAs, as in [38,40,74] or in C/C++ as in [75]. Through specific implementation, the hardware can be used optimally. Therefore, this approach has the highest potential. However, the implementations cannot be used for different hardware, tasks, and models. Another possibility to make a model edge applicable is quantization, Refs. [56,76], or lowering the sampling frequency or frame rate, Refs. [39,44,69]. Lowering model size while keeping high accuracy is a challenge that the authors of [77,78,79,80] made the main topic of their paper. Researchers and companies are making an effort to facilitate inference at the edge by offering optimization methods to compress ML models. Ref. [76] uses Model Optimizer Open VINO, Ref. [81] relies on LiteRT (formerly TensorFlow Lite), and [20] use TensorFlow Lite Micro.

It is worth giving more detailed information about the realization of edge applicability of the following systems. The authors of [39] propose a pulsed single-frequency ultrasound-based solution for object localization with one sender and eight receivers. The Texas Instruments TMS320F28379D microcontroller is used to implement the entire processing pipeline. Bandpass filters, window assessment, and thresholds are used in the processing. Beamforming and DOA estimation lead to successful localization of several objects. Another ultrasound-based HGR that can be trained in a neuroshield is presented in [82]. It requires one sender and one receiver and is based on an ultrasound pulse of a single frequency. The small gesture classification algorithm can be achieved, as the pre-processing includes relative ToF calculation. Paper [23] suggests a solution for HGR based on Infineon BGT60TR13C 60 GHz FMCW radar with one transmitter and three receivers. To prove edge applicability of the proposed algorithms, they are implemented on a Raspberry Pi4 with and without an Intel Neural Compute Stick 2. The algorithm is an optimization-based meta-learning approach. That means the main goal is to train the network to find a low-dimensional representation of the input that provides a basis for classification on new data—in the case of HGR, on user defined gestures. In [23], this representation occurs on the basis of RTI, RAI, and RDI, with VAE and a dense network or with a CNN. Ref. [77] captures data with TI IWR1443 76-81 GHz FMCW radar with three transmitters and four receivers. A Temporal Graph K-NN algorithm is generated on three radar point cloud data sets. Inference is implemented on a Raspberry Pi4, proving the edge applicability of this approach. The authors of [78] use Actioneer A1 pulsed radar. For classification, CNN-based automated feature extraction from RFDM is followed by a TCN. The final solution presented in [78] is specifically tuned for low power usage and memory footprint. It is implemented to run on a RISC-V microcontroller, the GAP8 from Greenwaves Technologies. Ref. [79] presents a transformer structure to replace the TinyRadar network, trained on the same data set. It outperforms the [78] solution in accuracy, power consumption and latency. Ref. [80] improves the edge applicability of the transformer presented in [79] by presenting a framework for edge transformers leveraging the RISC-V instruction set, applicable in several MCU classes. One possibility for showing edge applicability is by doing an HIL test. This was done for FMCW radar HGR in [75]. One of the earliest HGR based on WiFi [74] already introduces a real-time system. The output of this system is the calculated doppler shifts. An explanation is given how the four coarse gestures detected differ in their doppler shifts.

**Table 5:** Comparison of algorithms used for HGR with focus on real-time.

Classification Algorithm	Data type	Accuracy	Size	Inference Time	Effort for Edge Applicability
TCN [43]	Ultrasound	95.8%	Area ≤ 13,829 μm^2^ (analysis based on High-Level Synthesis (HLS))	Latency ≤ 302,945 cycles (analysis based on HLS)	Data flow transformation with FIR filter, sharing of hardware units
Beamforming, peak detection for distance estimation [38]	Ultrasound	FOV: 1 m, 90°; angular error 40 mm, 4°	Runs on a FPGA	Real time	Specific FPGA implementation
Traditional signal processing [46]	Ultrasound	Radial distance error: ±1.5 mm, 2°, arc error ±3.5 mm, ±3°	—	0.033 s	No ML/specific implementation
Tree-ensemble [47]	Ultrasound	96.7%	—	11.7 ms, incremental sample process time: 37.9 ms on laptop	Requires laptop, furhter adaptiations suggested by authors
TCN, beamforming [44]	Ultrasound	92–100%	10k parameters	270 k MAC operations per inference	Lower frame rate suggested
Amplitude envelope, B-Scan (horizontal sectional image), 3D-Scan (3D volume image) visualization with OpenGL (no HGR) [40]	Ultrasound	Lateral resolution: 11°, axial: 171.5 mm	Processing on a GPU	43 FPS (hemispherical pulse), 0.76 FPS (transmit beamforming) method for B-scan, 29 FPS (hemispherical pulse) method, 0.015 FPS (transmit beamforming) for 3D-Scan frame rate	Requires FPGA and GPU
Threshold based [1]	Image data	96%	Cognex 4400 vision processor and Sun 4/330	0.1 s	Special-purpose image processing computer used
Traditional signal processing for 1D tracking [83]	Ultrasound	Up to 2.5 mm	Implemented on a phone	2.76 ms	Specific implementation
Convolution-based [76]	Radar	78–99% depending on topology	Implemented on Intel Neural Compute Stick 2; 386,614 parameters	<1.013 s	Model Optimizer Open VINO, FPS32, FPS16
Convolution-based [81]	Radar	>99%	0.14 MB	0.147 ms with caching, 0.909 ms with caching	Lite Runtime (LiteRT), formerly TensorFlow Lite
Convolution based [63]	WiFi	96.03%	363,655 parameters, 195,239 parameters in compressed model	0.19 s	Linear mapping instead of convolutional layers
State space representation, angle-range features, clustering [73]	Radar	98.2%	—	3.84 s per gesture	Lower number of features by avoiding redundant ones
Range-doppler and micro-doppler with convolution based classification [56]	Radar	Cross user accuracy: 98.35%	15 KiB	9.062 ms on STM32F746G-DISCO	8-bit quantization
Combination of apps for hand tracking [84]	Vision	Goal: hand tracking	3 RPis used	34 FPS	Specific implementation
DTW, similarity measure [33]	LTE	No-ML system, no concrete evaluation of accuracy	Implemented on a phone	—	Specific implementation
SNN with liquid state machine (LSM) to create a spike train evaluated with logistic regression, RF, SVM [85]	Radar	98% 10-fold-cross validation	460 neurons (but 153 neurons already yields good results)	—	SNN, LSM encoding architecture for low processing requirements

**Table 6:** Comparison of algorithms used for HGR with focus on real-time systems that have actually been tested in real-time.

Classification Algorithm	Data Type	Accuracy	Size	Hardware of Online System	Inference Time	Energy Consumption of Inference	Effort for Edge Applicability
ShuffleNetV2 with GAN generated synthetic data [24]	Sound of finger sliding on surface	92.3%	34 k parameters	Android application on phone	—	—	Specific implementation
RNN [20]	Radar	98.4%	120 kB RAM, 278 kB flash	32-bit PSoC 6 Arm Cortex-M4	33% CPU time	75 mW	TensorFlow Lite Micro, 32-bit single precision floating point numbers
LSTM [69]	Radar	99.10% known participant, 98.48% new participant	—	Laptop	—	—	Lower sampling frequency, fft size, extraction of motion profiles
Traditional signal processing, Doppler effect [53]	Ultrasound	94.67–94.33%	—	Laptop	25.0 ms for pre-processing, ML model processing optimised by parallel processing and buffering	—	Specific implementation
Range-doppler, traditional signal processing [52]	Radar, depth image, sensor fusion	X, y, z direction error: 13.8, 10.8, 9.7 mm	—	TI Tiva Cortex M4F microcontroller	31 ms	<1 W by the radar—no further info	-
Range-doppler, convolution based DNN [86]	FMCW Radar, RGB, depth image, sensor fusion	94.1 (leave one session out cross-validation)	—	Quadro 6000 NVIDIA GPU	52 ms	1 W for the radar + 2.5 W for the imaging sensors; imaging sensor only used when gesture detected	-
Analog signal processing, beamforming, traditional signal processing [39]	Ultrasound	0.73–1.04% error	64.70 kBytes	Low cost MCU	20–45 Hz	—	After fft only required f-bins further processed
MLP, radial basis activation function (Neuroshield), desicion tree on ToF [82]	Ultrasound	92.87%, 98.4%, 96.94%	23 KB, 136 KB, 273 KB	Infineon XMC4700, improved speed when General Vision, Neuroshield device added	7.42–18.3 depending on window size	—	High compression of data during pre-processing (ToF basis for classification)
Optimization-based meta-learning [23]	Radar	1-shot: 84%, 3-shot: 92.6%, 5-shot: 94.2%	—	On Intel Neural Compute Stick	55–287 ms	Minimum: 5 mW per radar duty cycle	Two solutions: dimensionality reduction with Conv-VAE or convolution layers for feature extraction
Temporal graph kNN and attention based MPNN [77]	Data type	98.71%	1.56 parameters	RPi4	0.4 s (batch size 16)	—	Specific type of NN
2D Convolutional neural network, Temporal convolutional network [78]	Radar	86.6%	46,000 parameters; 92 KB	Parallel ultralow power processor	Up to 175 Hz tested	21 mW (for real-time prediction), 120 mW (system-level)	AutoTiler tool generated optimized C Code for parallel execution of the model
Transformer [79]	Radar	77.15%	263 kB	GAP8 or STM32L4, STM32H7	9.24 ms	0.47 mJ	Novel set of execution kernels for execution on MCU-class RISC-V and ARM Cortex-M cores
Transformer (Multi-Head Self-Attention) for mcu: Tiny Transformer [80]	Radar	Goal: deploying transformer models on commercial AI accelerators	5.19 MACs per cycle, 34 K cycles	GAP9	0.14 ms	4.92 uJ	Novel library of kernels with novel Multi-Head Self-Attention (MHSA) inference schedule and Depth-First Tiling (DFT) scheme for MHSA; for multi-platform deployment of encoder Tiny Transformers on mcus
CNN, LSTM, AoA [75]	Radar	93.11%	Less than 109 MB	Not yet implemented - HIL tests to verify real-time inference	Approximately 33 ms	—	Specific HAD (hand activity detection), specific implementation in C/C++ with CNN supported by TensorRT by NVIDIA
Doppler evaluation [74]	WiFi	No ML system, no concrete evaluation of accuracy	—	Laptop (Windows 7, Intel Core i7-3940XM 3.2 GHz)	25 ms	—	Specific FPGA implementation
ToF, beamforming [36]	Ultrasound	0.42 mm error at 0.5 m distance	—	—	5.9 ms per frame	—	2 digital beamformers (for x and y direction) no classification

## 9. Advantages of ML in HGR Systems

Since the beginning of HGR systems, ML algorithms have played a significant role, even though simple pre-defined gestures can be recognized without it. Every HGR application needs to be examined separately to determine whether an ML method is necessary or if doing so would merely cause overhead work. The requirement of a large, diverse data set and the search for adequate algorithms must not be underestimated. ML permits to add new gestures, makes the system more adaptable to new environments and users. Furthermore, ML enables the system to learn to recognize new gestures. The current research focus is on reducing the required amount of training data, inference time and processing requirements. A convenient, robust solution has not been found yet.

## 10. Evaluation of HGR According to Focus Areas

The evaluated papers have different focus areas. Resolution, real-time capability, continuous learning, robustness test, and solutions without ML are discussed in more detail in the following.

### 10.1. Focus Sensor Fusion

All reviewed modalities have advantages and drawbacks as summarized in Table 4. Combining different sensor types with sensor fusion for hand gesture classification leads to improved robustness and accuracy according to [52,86,87,88,89,90]. Table 7 compares approaches that implement combinations of several sensor types. Only [52] works without vision data and relies on depth data to evaluate the accuracy of radar data and for calibration. Two of the revised systems are implemented as real-time systems. The majority of systems, Refs. [88,89,90], use an online data base that provides skeleton data points. The authors of [87,89] compare several different fusion structures. Ref. [89] found that score-level fusion resulted in highest accuracy. In this case, the scores of classification on skeleton data and of depth data are combined by calculating their average. Since both sensor types are initially processed individually and the classification result is combined, this can be seen as late fusion. The authors of [87] also found the best results when combining the information from different modalities late in the classification process. More specifically, radar, optical, and skeletal data are first separately classified. The output of these classifiers are vectors that contain a score for each possible gesture relating to the probability that this gesture is the performed one. In a subsequent step, these vectors are further combined and processed by another network. The output of the second network provides higher accuracy values. The authors of paper [86] pre-process depth and RGB data individually, but use depth data to extract angular velocity values from the radar data only in the region of the moving hand. In their evaluation, they compare accuracies from classifying each of the modalities individually with classifying the joined input from all modalities. If all modalities are used, the input of the network already contains all information. This approach therefore relies on early fusion.

The majority of research up to the present relies on HGR based on one sensor type, but the results of the available research on sensor fusion approaches for HGR show increased robustness and accuracy through sensor fusion. The reason for this effect is probably that different sensors complement each other. The drawback of one sensor can be made up for by using another that is especially well suited to make up for this drawback. Table 4 can serve as a starting point for selecting modalities for optimal combinations. It must be mentioned that sensor fusion models require several sensors and thus more—and potentially bigger—hardware, power, and processing requirements. It follows that sensor fusion is a good way to improve the robustness of an HGR system. Therefore, sensor fusion is recommended for systems that require high reliability. Simultaneously, it may not be the right choice in cases where the main focus lies on reducing system size or processing and power requirements.

### 10.2. Focus Resolution

For some HGR systems, the focus lies in resolution. These systems are summarized in Table 8. Of the approaches considered, that was the case only for either radar or ultrasound-based ones. Of the applications for radar, one approach involved sensor fusion of radar and depth image for improved hand tracking. In the case of [91], resolution is seen as a factor to be improved by feature search, which is the main topic of the paper. Ref. [92] focuses on improving angular resolution, which is achieved by adding the complex values of the Fourier transform to the information fed into the evaluating neural network. The ultrasound-based solutions with a focus on resolution showcase the possibilities that airborne ultrasound holds for imaging. Ref. [41] goes furthest, using 64 transducers.

### 10.3. Focus Real-Time

There are two types of real-time systems evaluated in the scope of this work. On the one hand, there are algorithms evaluated for their real-time capability on the basis of a data set, as summarized in Table 5. On the other hand, there are systems that propose an end-to-end HGR system including the sensor device and pre-processing, such as the ones in Table 6. Both types of systems can be evaluated with regard to latency, as well as hardware and power requirements. Systems that are not based on ML sometimes do not evaluate their algorithm concerning accuracy on a data set. Sometimes, the size of an algorithm is stated in parameters of the ML model or the size of the algorithm in kB memory used. Furthermore, it is difficult to evaluate a system’s latency. Some papers provide speed information on the basis of high-level synthesis. Others state the required processing time, including the hardware used for processing. But timing depends on parameters, number of operations, size of input data, method of memory access, method of caching, and hardware parallelization. In paper [80], lots of specific information is given for hardware-optimized transformer models. It specifically compares possible optimizations for three different microcontrollers and compares them.

### 10.4. Focus Continuous Learning

Few algorithms implement ways for continuous learning during runtime in their solution, such as those in Table 9. On the ML algorithm side, incremental training, zero-shot, 1-shot, few-shot, and re-training with new unlabeled data are possibilities. Ref. [44] suggests compound gestures to increase the number of hand gestures. Zero-, 1-, and few-shot options are mostly used to increase the vocabulary of the system, while re-training has a focus on improving the recognition robustness of known gestures.

### 10.5. Focus Robustness

There are several levels of robustness that can be tested, as Table 10 shows. Testing can be in regard to device and setup. Two sensors of the same type may have slightly different behavior, which can affect the accuracy of a trained model. If several devices are required for an HGR system, the positioning of the devices has an influence. As humans make gestures in slightly distinct ways and hand sizes vary, cross-user robustness needs evaluation. Furthermore, the environment a system is placed in has an influence on it. Rarely, authors of a paper about HGR try to determine what influences the system. Most systems are tested in an indoor environment.

### 10.6. Focus No-ML

Several systems successfully use ML-free signal processing techniques, like those in Table 11. These systems can be tested—as in ML—with a data set. Many of the systems target imaging or tracking, which is more difficult to test. In those cases, resolution is a measure for the system performance.

### 10.7. Other Innovative Solutions

Some innovative solutions do not fit any of the previous focus areas but are nevertheless worth mentioning here. They are summarized in Table 12. The application [71] stands out by using a plate to generate ultrasound waves. Refs. [27,70] set their research focus on sparsity. The former uses sparsity even in the signal-generation process, which leads to improved applicability on edge devices in the long run due to lower processing and power requirements. Ref. [35] research imaging with WiFi. An almost entirely unexplored field up to now, Ref. [93] introduce a baffle system for ultrasound image generation. Several solutions show a sensor fusion approach, such as [25,87,87]. Here, a major research question is where in the classification process the data fusion should take place. Refs. [19,94] introduce purely ML-based solutions that omit the Fourier transform in pre-processing. Ref. [30] introduces a new framework for radar pre-processing for HGR. Refs. [25,29] show that online-available deep learning algorithms are adaptable for HGR. Ref. [21] introduces a meta-learning approach that is potentially usable for novel, adaptable hand gestures as it is based on the idea of learning to compare and select the closest gesture as a result. Ref. [64] is the only solution for small-scale movement classification with WiFi.

While it is important to test and publish about new approaches and algorithms capable of HGR, it is difficult to determine the usability of such algorithms in edge devices. Further research is needed to understand the requirements of each of those algorithms and whether their unique attributes advance the usability of HGR systems as convenient, everyday HMI on the consumer side and in industry.

**Table 8:** Comparison of algorithms used for HGR with focus on resolution.

Classification Algorithm	Data Type	Real-Time System	Number of Sensors	Resolution	Special Feature
Range-doppler, traditional signal processing [52]	Radar, depth image	Yes, 32 fps	4 radar antennas, 1 depth camera	x, y, z direction error: 13.8, 10.8, 9.7 mm	Sensor fusion
Traditional signal processing, beamforming [41]	Ultrasound	2 min per image	64 Murata MA40S4S transducers	2.3° angular resolution	Goal: high precision imaging
Beamforming, peak detection for distance estimation [38]	Ultrasound	Yes	1 transmitter, 7 receivers	1.67 cm, 1.5°	Creates a B-mode image
Traditional singal processing [46]	Ultrasound	Yes, 4.4 ms at maximum range	1 transmitter, 7 receivers	Radial distance error: ±1.5 mm, 2, arc error: ±3.5 mm, ±3 deg	Thin-film aluminum nitride transducers, creates a B-mode image
Amplitude envelope, B-Scan (horizontal sectional image), 3D-Scan (3D volume image) visualization with OpenGL (no HGR) [40]	Ultrasound	Yes	64 Murata MA40S4S transducers	Lateral resolution: 11°, axial: 171.5 mm	Creates B-Scans
Traditional signal processing for 1D tracking [83]	Ultrasound	Yes, 2.76 ms per 10.7 ms audio segment	1 transmitter, 1 receiver	Accuracy up to 2.5 mm distance change, 3.6 mm path length change, 2.1 mm trajectory tracking error	Continuous wave, implementation on a phone
Complex-valued neural networks for DOA angle estimation [92]	Radar	No	4 transmitters, 4 receivers	Mean absolute error less than 4°	Processing of complex values
Multi dimensional dynamic time warping of 60 features found with quantum-inspired evolutionary algorithm [91]	Radar	No	1 transmitter, 4 receivers	2.7 cm, 2.2 cm/s	Quantum-inspired evolutionary algorithm
CNN [95]	Ultrasound	No	1 transmitter, 1 receiver	Doppler f: 15.6 Hz, velocity: 9 mm/s, range 6.9 mm, unambiguous distance: 0.172 m, unambiguous velocity: 0.2867 m/s	Spectrogram/micro Doppler based features used for classification
Channel Impulse Response, convolution based classification [60]	Ultrasound	Yes	2 speakers, 1 to 4 microphones	7 mm	Implementation on a phone; impact of number of microphones studied
ToF, beamforming [36]	Ultrasound	Yes, 170 fps	1 transmitter, 7 receivers	0.42 mm error at 0.5 m distance	Creates a B-mode image

**Table 9:** Comparison of algorithms used for HGR with continuous learning.

Classification Algorithm	Data Type	Accuracy	Real-Time System	Continuous Learning	Stability in Different Environments
Tree-ensemble [47]	Ultrasound	96.7%	Yes	Incremental training	Cross-user
TCN, beamforming [44]	Ultrasound	92–100%	Yes	Compound gestures	-
Convolution based, training enhanced with unsupervised learning [66]	Radar	96.72%	No	Possible with new unsupervised data	6 different indoor locations
3D CNN, meta-learning [96]	Radar	5-way, 5-shot: 98.10% 5-way, 1-shot: 98.40%	No	No, but 1-shot learning possible	-
One-shot calibration, template-based recognizer [97]	Radar	Up to: user dependent: 96.1%, user independent: 31.5%	No	Few-shot options	All tests behind either wood, PVC, or glass
Similarity measure [98]	Ultrasound	Only one sample for training: 93.97%	No	Goal: use as few data as possible, uses supervised and unsupervised learning to leverage unlabeled data as well as possible	-

**Table 10:** Comparison of algorithms used for HGR with focus on robustness.

Classification Algorithm	Data Type	Accuracy	Real-Time System	Stability in Different Environments
CNN-Transformer [55]	Radar	Up to 98%	No	Interference by a cylindrical object and a non-recognizable hand gesture
LSTM-CTC (Connectionist Temporal Classificaton) [99]	Radar	96%	No	Robustness test regarding distance, scale of hand gesture, duration time of hand gesture
LSTM [69]	Radar	99.10% known participant, 98.48% new participant	Yes	99.10% known participant, 98.48% new participant
Traditional signal processing, Doppler effect [53]	Ultrasound	94.67–94.33%	Yes	Home, Cafe (accuracy respectively)
FFT normalization, squared continuous frame subtraction, Gaussian smoothing, Doppler effect [54]	Ultrasound	up to 96 %	Yes	Tested in 2 indoor environments, in a bus
ShuffleNetV2 with GAN generated synthetic data [24]	Sound of finger sliding on surface	92.3%	Yes	3 indoor scenarios tested
Tree-ensemble [47]	Ultrasound	96.7%	Yes	Cross-user
Fresnel zone calculation, decrease and increase in reflection path length [32]	LTE	98 %	No	3 environments, indoor and outdoor
Convolution-based feature extraction(SonicGest-Net: convolutions and 2 residual blocks as suggested in ResNet), GAN with Wasserstein distance based on gradient penalty for example generation [65]	Ultrasound	98.9%	No	2 indoor environments
Based on signal change primitives, thresholds [34]	WiFi	87.5% with one access point, 96% with 3 access points	No	2 indoor environments
SVM [100]	WiFi	88–92% depending on scenario (non line of sight, line of sight respectively)	No	2 indoor environments
Wavelet analysis and dynamic time warping [101]	WiFi	97.3–91.8%	No	5 indoor scenarios
Phase difference matrix, modified attention-based bi-directional gate recurrent unit classification, sparce recovery for automated feature extraction [102]	WiFi	96%	No	96% in two indoor environments
Time reversal resonating strength, angle and intersections b/w segments [103]	WiFi	92%	No	“Certain” amount of environmental shift
CNN, transformer encoder [62]	WiFi	In domain: 96.78%, cross domain: 88.27%	No	Cross domain means cross-user in this paper
Attention based [104]	WiFi	In domain: 99.69%, cross-location: 96.95%, cross-orientation: 93.71%	No	In domain, cross-location, cross-orientation tests
AlexNet (convolution based deep learning) [105]	Ultrasound	92.9%	No	Stable for different time per gesture; difficulties with different phone placement and distance
Traditional signal processing for 1D tracking [83]	Ultrasound	up to 2.5 mm	Yes	Sensitive to environmental changes like wind and angles
Principal component analysis [106]	WiFi	93%	No	Two indoor environments tested
K-nearest neighbor, SVM [107]	Ultrasound and audio	77.8% for combination of audio and ultrasound	No	Cross user accuracy drop even in same environment
CNN-TCN [107]	Radar	98.61 % in original environment, 97.22% in new environments	No	97.22% accuracy in new environments
Convolution based, training enhanced with unsupervised learning [66]	Radar	96.72%	No	6 different indoor locations
Domain independent angle-dopplear maps, attention based NN [108]	Radar	In-domain: 98.9%, cross-domain: 85.6–97.4%	No	Cross-domain means other indoor environments and other users
Convolution based, transfer learning [109]	Radar	98%	No	Transfer from uwb radar data set to fmcw radar data set
State space representation, angle-range features, clustering [73]	Radar	98.2%	Yes	2 different radar housings tested
One-shot calibration, template-based recognizer [97]	Radar	User dependent: 96.1%, user independent: 22.8%	No	All tests behind either wood, PVC, or glass
Convolution-based combined with gate-based classification [67]	Radar	Up to 99% for single target in different environments, 93% for dual target	No	Tested in new environments
Convolution based [61]	Radar	98.75%	No	Proved well suited for different hand angles
Range-doppler and micro-doppler with convolution based classification [56]	Radar	Cross user accuracy: 98.35%	Yes	Cross user accuracy: 98.35%
Spiking neural network [61]	Visison	77%	No	2 online data sets used
CSI [31]	5G (doppler)	94–95% for 100 MHz bandwidth, 70–75% for 20 MHz bandwidth	No	Evaluation of impact of bandwidth, evaluated in different scenarios
Domain independent features, distributed model training, federated transfer learning [110]	WiFi	In domain: 99.6%, cross domain: 93%	No	In and cross domain tests

**Table 11:** Comparison of algorithms used for HGR without ML.

Classification Algorithm	Data Type	Accuracy	Real-Time System	Special Feature
Traditional signal processing, Doppler effect [53]	Ultrasound	94.67, 94.33%; false motion event detection: 2.5/min, 6/min Home, Cafe (accuracy, false mortion event detection, respectively)	Yes	Only hardware required is a laptop
Range-doppler, traditional signal processing [52]	Radar, depth image, sensor fusion	x, y, z direction error: 13.8, 10.8, 9.7 mm	Yes, inference time: 31 ms	Sensor fusion radar and depth image
Threshold based velocity feature based comparison [50]	Range images	“Gestures are appropriately recognised”	No	From 1998; one of the earliest approaches
Thresholds, Matching of magnitude and direction for each finger [51]	Vision	“Almost perfect”	more than 24 s per gesture	From 1994; one of the earliest approaches
FFT normalization, squared continuous frame subtraction, Gaussian smoothing, Doppler effect [54]	Ultrasound	up to 96%	Yes	Only hardware required is a phone, tested in a bus
SSL (MUSIC), beamforming, cross-correlation [42]	Ultrasound	Angular resolution 3°	No	Goal: imaging, posture estimation rather than HGR
Traditional singal processing [46]	Ultrasound	Radial distance error: ±1.5 mm, 2°, arc error ±3.5 mm, ±3°	Yes	Goal: beamforming based, result is a b-mode image
Amplitude envelope, B-Scan (horizontal sectional image), 3D-Scan (3D volume image) visualization with OpenGL (no HGR) [40]	Ultrasound	Lateral resolution: 11°, axial: 171.5 mm	Yes	Utilises a uniform 3D printed waveguide to reduce interelement spacing between receivers for gating lobe-free beamforming, result is a b-scan image
Fresnel zone calculation, decrease and increase in reflection path length [32]	LTE	98%	No	3 environments, indoor and outdoor
Threshold based [1]	Image data	96%	Yes	Even user dependent
Based on signal change primitives, thresholds [34]	WiFi	87.5% with one access point, 96% with 3 access points	No	2 indoor environments
Traditional signal processing for 1D tracking [83]	Ultrasound	Up to 2.5 mm	Yes	Sensitive to environmental changes like wind and angles
Principal component analysis [106]	WiFi	93 %	No	Two indoor environments tested, only required hardware is a smartphone
Combination of apps for hand tracking [84]	Vision	Goal: hand tracking	Yes	Goal: reach real-time capability, generation of a stick model of a hand
ToF, beamforming [36]	Ultrasound	0.42 mm error at 0.5 m distance	Yes	Beamforming based, goal is b-mode image

**Table 12:** Other innovative solutions for HGR.

Classification Algorithm	Data Type	Accuracy	Special Feature
RDFrame from UWB radar [30]	Radar	96 %	Novel framework for UWB radar to RDF; tested NN combinations: 3D CNN - FCNN, 3D CNN - k-NN, 3D - SVM, 2D CNN - LSTM
GoogLeNet inspired, convolution based, inception modules [29]	Radar	95%	Based on GoogleLeNet
Cross-correlation, inverse Fourier Transform [35]	WiFi	recognition of an “X” of 94 × 94 cm	Image reconstruction with WiFi
SVM (walking person, two people walking, moving wheeled vehicle) (no HGR) [28]	Radar	76.3–94.2% depending on architecture and 1- or 5-shot experiment	Classification for ground moving targets: single person, two persons, vehicle
Google Xception Model, Convolution based [25]	Ultrasound	93.58%	Data augmentation, different fusion architectures evaluated
Nearest Neigbor, modified Hausdorff distance [27]	Radar	96%	Sparcity driven method
Meta learning, comparison module, weighting module [21]	Radar	95% For 3-shot, 5-way	Embedding module with training to learn to compare
CNN, LSTM, desiciton tree [64]	WiFi	97.57%	Focus on small scale motions with WiFi
Convolution-based [59]	Radar	99%	Evaluation of benefit of phase information
GRU [71]	Ultrasound	93.5%	Use of a thin plate for ultrasound signal generation
SNN beamforming, GRU classification or quantized spiking NN [70]	Ultrasound	86 %	Spike based beamforming approach
3 complex linear layers in relation to range, doppler, and angle extraction with DFT + convolution based classifier [94]	Radar	98.06%	Suggestions to replace DFT in pre-processing
convolution-based from time-velocity—and time-angle diagram XGBoost and LSTM binay classifiers compared [111]	Radar	95%	Focus on micro gestures
Attention based [87]	Vision, radar	—	Fusion of radar and optical data; evaluation of early, intermediate, and late fusion and late and intermediate attention mechanisms
Support Vector Machine [93]	Ultrasound	92%	Introduction of bio-inspired baffles to ultrasound imaging in air

## 11. Latest Developments in HGR

The latest HGR solutions target enhanced cross-user, cross-device, and cross-environment robustness. To increase robustness, different types of deep learning methods are combined. Meta-learning, few-shot learning, ensemble learning and semi-supervised learning are processes being explored. Furthermore, information from several types of pre-processing, RDI, RAI, DAI, and micro-Doppler spectrogram are combined and processed in parallel. Temporal and spatial information are fused to improve robustness of the system. CNNs for example, which do not process temporal information, are combined with neural networks used to leverage temporal information. CNNs can only perform gesture classification if they are fed the whole gesture at once. Therefore, different gesture speeds pose a problem and the classification process can only be initiated after the full gesture has been carried out. The lack of time-relevant data can only be compensated for by additional algorithms for parallel extraction of time-related information. TCN uses dilated convolutions to extract time-related information.

Other algorithms like RNN, such as LSTM and GRU, as well as Transformer structures like ViT and CrossViT, extract time-relevant information using memory. In the case of RNN, this is done through back-propagation. This works well for leveraging information in the close proximity of the processed piece of data but causes exploding and vanishing gradient problems for information temporally further apart. LSTM and GRU use gating mechanisms to extract relevant, temporally close and far information. Transformer structures use attention mechanisms to extract time-related information.

Other recent approaches work towards lowering the system and inference time requirements by lowering processing demands and sensor needed. One possibility for moving training from HCP platforms to the edge is via federated learning. It is further beneficial during runtime, especially for robustness and versatility of the system. This can be done with continuous learning. Alternatively, the data gathered during runtime must be sent to a HPC platform for retraining. In all cases, the incoming new data is likely to be unlabeled. Therefore, at least a preliminary evaluation must rely on unsupervised learning—for example, a clustering algorithm—to predict a pseudo-label.

Zero-, one-, and few-shot learning can support cross-user and cross-environment robustness.

On the basis of meta-learning, the data set can be expanded and training time reduced.

## 12. Innovative Theories

Innovations in HGR can start at different points. For ML-based solutions, novel processes such as meta-learning, continuous learning, and ensemble learning open doors for improvement of adaptability of HGR systems. Meta-learning is expected to allow learning of new gestures. Continuous learning will probably improve accuracy when a system is placed in a novel environment and adjust it perfectly to one or several specific users. Ensemble learning distributes the training processing load across multiple hardware nodes, thereby enhancing learning directly at the network edge. However, even traditional training can be used for continuous improvement of a system by connecting the system to a cloud, where reiterations of training can improve the system while it is already employed. In summary, the trend is anticipated to go towards adaptation of systems during their application phase through ML. The expectation is that this will increase the robustness of the system. In cases where continuous system improvement is not possible, as when it is implemented in an isolated working environment, sensor fusion or architecture fusion can be the way to go. Each sensor technology comes with specific advantages and drawbacks, as summarized in Table 7. Data combined from several sources, therefore, can complement each other, leading to improved HGR. In the same way, different ML architectures are often especially good at a specific task(s) while being less suitable for others. An overview of the advantages and disadvantages of classification algorithms is provided in Table 3. By combining several architectures, the unique strengths of each are leveraged to refine the final outcome. Lastly, especially for simple tasks or in cases where the application environment is completely defined from the start, solutions without ML should continue to be considered. The reason is that algorithms without ML are, to date, in most cases the most efficient in processing requirements and timing.

## 13. Potential Future Applications

Automation continues to enter more and more parts of our life. But even nowadays, some devices come with complicated input modalities. HGR is a possible step towards lightweight, simple, intuitive input systems even for very small devices. Ultrasound-based systems, for example, can be integrated into smartphones or headphones, as the required hardware consists of loudspeaker and microphone. Radar-based systems may be best suited for in-cabin sensing or other cases where privacy protection is crucial. WiFi, 4G, and 5G may implement HGR as an additional feature when the system is already in place. Vision is expected to be used for most fine-grained gestures but probably in combination with another modality, as vision alone does not receive often-crucial depth information.

## 14. Conclusions and Outlook

For WiFi, radar, and ultrasound, the sent pulse–frequency modulation can increase SNR and facilitate echo extraction. Signal strength and intensity distribution can be used for evaluation on the receiver side. Following vision, radar is the most researched sensor type for HGR. To allow 3D gestures for vision-based systems, a depth camera or stereo vision technology is required to enhance images with depth information.

The goal of pre-processing is to lower the size of the input for classification. This requires reduction of noise and data that does not contribute to classification while keeping useful parts. This increases information density in the data. A possible way of removing noise and unnecessary data is using given information about the physics of hand gestures such as the expected size of the hand, speed, and location of the hand. These properties are the same for all sensor types.

Table 13 summarizes advantages and drawbacks of pre-processing algorithms in the time domain. Time domain-based algorithms avoid information loss and time delay through the Fourier transform. It is possible to apply no pre-processing to the input information. This ensures that no data loss occurs before processing. However, the complexity and size of neural networks depend on the size and complexity of the input data. Thus, in the case of no pre-processing, the neural network is more complex and requires more training data and time. Creating an intensity map does not shrink the amount of input data significantly. However, the majorityt of neural networks for image data evaluation can be used on intensity maps. TGC is a simple signal-processing method applicable in combination with other pre-processing algorithms. It assures that echoes from farther-away objects are amplified. In theory, it thereby enhances all following processing steps. Sometimes, it is not clear how much a signal is attenuated along its path, as that depends on environmental influences. Therefore, it can be difficult to apply TGC correctly, and applying it wrongly can lower data quality. Cross-correlation and wavelet transform both enhance signal parts that look similar to an input signal. The main drawback of such pre-processing is that noise that is similar to the input signal is also enhanced and can cause misinterpretations in the classification algorithm. RSS and CSI reveal information about the status of a transmitted signal and its strength. CSI is more recent and difficult to process but contains more granular and precise information. Those techniques are originally used in WiFi processing but can be adapted for other signal types.

Table 14 outlines advantages and drawbacks of pre-processing algorithms in the frequency domain. The majority of pre-processing algorithms do Fourier transform. Often, pre-processing with and without Fourier transform are combined. In radar, usually one or several of the maps RAM, RDM, or ADM are used. Each of them requires quite some processing, and the result is not easily understandable. However, visualizations of those maps work well with image processing algorithms. If it is clear beforehand which information, range, frequency shift, or angle is the most relevant one, processing can be done only on the corresponding map. More fine-grained evaluation is often based on micro-Doppler/spectrogram maps. When processing any type of maps, we face the challenge that gestures are carried out at different speeds. Possibilities to cope with that are dynamic speed or time warping. However, these require substantial processing power. Sending a specific frequency pattern and searching for it in the received data, for example with wavelet transform or cross-correlation, can avoid unintentional enhancement of noise and false echo detections. Beamforming on the sender side increases the SNR as it decreases the width of the sent beam and increases its intensity. However, sender-side beamforming evaluates a lower area at a time. Therefore, the algorithm has to evaluate the received echo severally for different beam directions to analyze an area of similar size. Beamforming on the receiver side requires simultaneous processing of several directions. This entails the use of significant processing power but increases processing speed. Band-pass filtering enhances the signal in the regarded frequency range and removes unwanted frequencies from the signal. This can distort the signal but has mostly a strongly positive effect and requires little processing power. The classification methodology highly depends on pre-processing. The lower the amount of pre-processing, the larger the model size. Novel processes for ML enhance robustness, facilitate learning, and lower the amount of training data needed.

Table 3 compares classification approaches, including algorithms and processes for HGR. Independent of the sensor, HGR systems have some common goals, such as increasing the robustness of the system, ease of use, and ease of integration in an overall system or in an edge device. Usually, one of them is the main goal targeted by a novel solution presented in a paper.

A summary of main goals and possible solutions for further HGR research is given in Table 15. Most opportunities for improvement are seen in the robustness of touchless HGR systems. This includes making the system usable in different environments. Retraining and calibration may be a way to go. At the same time, these are feasible approaches to mitigate the impact of concept drift. The best case is generalizing the system sufficiently such that it works independent of surroundings, sensor, and user.

We are able to show that touchless HCI are worth further investigation. Taking into account the achievements in this field across different sensor types avoids repetition of mistakes. Furthermore, it underlines which methodologies have worked for one sensor and therefore are worth evaluating for another.

## Figures and Tables

**Table 1:** Challenges of HGR.

Challenge	Reason
Detection of one or more hands	Similarity between hand and background
	Different hand sizes
	Different location of hand at beginning and end of the same gesture
Tracking of a hand	Different hand speeds
Classification of a hand gesture	Variation of person: way of doing a gesture, speed, hand size, hand positioning towards the sensor
	Variation of background: noise, obstacles, moving objects

**Table 2:** Taxonomy to categorize hand gestures for improved evaluation of the complexity of the task.

Taxonomy	Meaning
Coarse vs. fine	Hand or arm gestures vs. finger gestures
Complex vs. simple	A gesture that contains at least one turn vs. a gesture that has only one direction

**Table 3:** Advantages and disadvantages of classification approaches for HGR.

Classification Approach	Advantages	Drawback	Special Feature
DTW	Supports different speeds	High computational complexity	Similarity measure for time dependent sequences
CNN	Extensively researched, successfully used on pictures	No time evaluation	Kernels, convolutions
TCN	Keeps information about time, less memory and training time required compared to LSTM, no exploding, vanishing gradient problem	Harder to adapt to meta and transfer learning	Input size equals output size, causal convolutions: all predictions are only based on past timesteps, dilated convolutions: wider convolution area with same computation cost
LSTM, GRU (RNN)	Good for time series data, no exploding or vanishing gradient problem	Big model size, many parameters, long and processing intensive training time	Gates for time related information
ViT, CrossViT (transformer)	Good for images and time series data, model size better than for LSTM, GRU, faster and less processing intensive training than GRU, LSTM	Large number of parameters, therefore need a large data set for training, long training time	Attention method for time related information
SNN	Small size, fast processing, low processing requirements	More difficult to train	Functionality inspired by neurones
Fusion architectures	Higher accuracy	Parallel processing required, higher processing load	Combines advantages of several structures in one
Random forest, decision tree (ensemble learning method)	Simple	May not be accurate enough especially for fine-grained features	Combinable
Meta-learning (zero-, one-, few-shot)	Lower training time, lower training data set required, for adjustable problems, fast adaptation to new problems	Forgetting of old problems	Adaptability; learning to learn; process of training a model or an algorithm to understand and improve its own learning process
Federated learning	Data privacy, decentralized training, heterogeneous data, suitable for edge applications	Reaching good overall accuracy is more difficult compared to other architectures	Training takes place in edge nodes, updates of the central model based on gradients or parameters obtained in edge training
GAN	Can be used to enhance the data set	Not appropriate for classification	Generates new data to create or enhance a data set
Ensemble learning	Merges predictions from several models	More complicated structure	Can be run on edge nodes
Reinforcement learning	Decision making agent, adaptive adjustment of signal processing, filter settings, and threshold levels	Exploration and exploitation ratio difficult with limited data	Adaptability over time

**Table 4:** Summary of advantages and drawbacks of different sensor modalities.

Modality	Advantage	Drawback
Vision	Fine grained Lots of information	No depth information Good lighting conditions required High data size High power consumption High processing requirements Big sensor size Privacy issues
Radar	Low power consumption Smaller sensor size Works in darkness	Less accuracy Susceptible to electro-magnetic noise
Ultra-sound	Lowest power consumption Smallest sensor size Works in darkness Low processing power needed	Low range Low resolution Susceptible to acoustic noise
WIFI, LTE, 4G, 5G	Hardware easily available Scalable	CSI or RSSI pre-processing required Very low accuracy High noise in most surroundings Often cannot work simultaneously with normal WIFI use for connection to the internet Higher power consumption

**Table 7:** Comparison of algorithms used for HGR with sensor fusion.

Classification Algorithm	Data Types	Number of Sensors	Result (Resolution or Accuracy)	Sensor Fusion Method	Goal/Advantage of Sensor Fusion
Range-doppler, traditional signal processing [52]	Radar, depth image	4 radar antennas, 1 depth camera	x, y, z direction error: 13.8, 10.8, 9.7 mm	Depth sensor is used for calibration	Evaluation of accuracy of the radar
Attention based [87]	Vision: RGB, skeletal data, radar	radar: 2 transmitters, 4 receivers	Accuracy: 0.986–1.0; best results with late merging with late attention	Fusion of radar and optical data; evaluation of early, intermediate, and late fusion and late and intermediate attention mechanisms	Radar modality is of complementary nature to RGB and skeletal data. Therefore, multi modal input leads to improved classification results.
Range-doppler, convolution based DNN [86]	FMCW radar, RGB, depth image	4 radar antennas, 1 depth camera including the RGB camera	Accuracy 94.1 (leave one session out cross-validation)	Data preparation for training, Evaluation of the sensors separately	improved accuracy, robustness, power consumption
Convolutional Recurrent Neural Network) CRNN [88]	Depth, 2D-skeleton points	Depth through stereo vision, RGB camera	80.00 to 96.43% accuracy	Sensor fusion at input (early fusion)	Improved accuracy, improved parameter efficiency
CNN, RNN [89]	Depth, 2D-skeleton points	Depth through stereo vision, RGB camera	85.46% accuracy	Fusion at three levels: feature - level, Score-level, decision-level	Improved accuracy
Attention-based [90]	Skeleton data, rgb, depth, optical flow image	Depth through stereo vision, RGB camera	—	Late fusion of skeletal data, early fusion of the other modalities	Improved accuracy

**Table 13:** Advantages and disadvantages of different pre-processing algorithms in the time domain for HGR.

Algorithm	Advantages	Drawback
No pre-processing algorithm	No information lost	No compression
Tgc	Negates effect of attenuation	Difficult to know required strength, adaptability to current situation only with calibration possible, may enhance far away objects too much - cause of multiple reflections
Hilbert envelope	Makes data more clear	May cause data loss
Cross-correlation	Supports finding the middle of an echo, enhances SNR	May not be sufficiently accurate (high output where no real echo)/ may cause output of wrong echo
Wavelet transform (may include frequency-domain components)	Supports finding the middle of an echo, enhances SNR, more flexible than cross-correlation	May not be sufficiently accurate (high output where no real echo)/ may cause output of wrong echo
Intensity map	Good visualization possible, can be used in combination with image processing	Little data compression
RSS	easy to obtain	Less granular and precise than CSI
CSI	Information about the channel state concerning scattering, fading, and power decay; channel state contains information about the environment, objects and changes of the environment	More difficult to obtain than RSS

**Table 14:** Advantages and disadvantages of different pre-processing algorithms in the frequency domain for HGR.

Algorithm	Advantages	Drawback
Fourier transform (micro-doppler/spectrogram), find an example in Figure 7	Evaluation of signal in frequency domain possible	May cause distortion of real data, either time or f-accurate
RAM	Extracts a combination of direction and distance information	May cause distortion of real data, complex computation for interpretation required
RDM, as shown in Figure 15	Extracts a combination of velocity and distance information	May cause distortion of real data, difficulty of separating several objects
ADM	Extracts a combination of velocity and direction information	May cause distortion of real data, complex computation for interpretation required
Send frequency pattern and search	Facilitates cross-correlation, higher SNR possible, less interference	More complicated to generate and process than a single frequency
Beamforming	Increased range and accuracy possible, less interference	Computationally expensive
Band-pass	Filter for only required f-spectrum	Not applicable for wide band actuation, may distort the signal

**Table 15:** Current goals of HGR system algorithms and promising solutions.

Goal	Promising Solution
Lower required inference processing	Compression of models through quantization, pruning, distillation, SNN
Increase accuracy	Parallel processing the output of several pre-procesing algorithms, usage of the imaginary part of the Fourier transform
Increase robustness	More diverse training data, data augmentation, generation of more versatile training data with GAN, increased data set with unlabeled data, incremental learning
Lower size of training data set	Data augmentation, generation of more versatile training data with GAN, zero-, one-, few-shot learning, meta-learning
Decrease inference time	Same as lower required inference processing
Increase gesture data set, adaptable gesture data set	Meta-learning, continuous learning
Lower training time and requirements	Distributed learning, meta-learning, use of pre-trained models

## Abbreviations

The following abbreviations are used in this manuscript:

HGR	Hand gesture recognition
MIMO	Multiple-input multiple-output
HMI	Human–machine interface
IoT	Internet of Things
ToF	Time of flight
DTW	Dynamic time warping
SFCW	Single-frequency continuous-wave
FMCW	Frequency-modulated continuous-wave
UWB	Ultra wide band
CNN	Convolutional neural network
RDM	Range doppler map
PRT	Pulse repetition time
RSSI	Received signal strength indication
CSI	Channel state information
OFDM	Orthogonal frequency division multiplexing
FM	Frequency modulation
GR	Gesture recognition
GAN	Generative adversarial network
ML	Machine learning
SNN	Spiking neural network
OMP	Orthogonal matching pursuit
NR	New radio
TCN	Temporal convolutional network
LSTM	Long short-term memory
GRU	Gated recurrent unit
TGC	Time gain compensation
RAM	Range angle map
RDM	Range doppler map
ADM	Angle doppler map
SNR	Signal noise ratio
RSS	Received signal strength
